# Allosteric Site on SHIP2 Identified Through Fluorescent Ligand Screening and Crystallography: A Potential New Target for Intervention

**DOI:** 10.1021/acs.jmedchem.0c01944

**Published:** 2021-03-16

**Authors:** Hayley Whitfield, Andrew M. Hemmings, Stephen J. Mills, Kendall Baker, Gaye White, Stuart Rushworth, Andrew M. Riley, Barry V. L. Potter, Charles A. Brearley

**Affiliations:** † School of Biological Sciences, University of East Anglia, Norwich Research Park, Norwich NR4 7TJ, U.K.; ‡ School of Chemistry, University of East Anglia, Norwich Research Park, Norwich NR4 7TJ, U.K.; § Department of Molecular Haematology; Norwich Medical School, University of East Anglia, Norwich NR4 7TJ, U.K.; ∥ Medicinal Chemistry & Drug Discovery, Department of Pharmacology, University of Oxford, Mansfield Road, Oxford OX1 3QT, U.K.

## Abstract

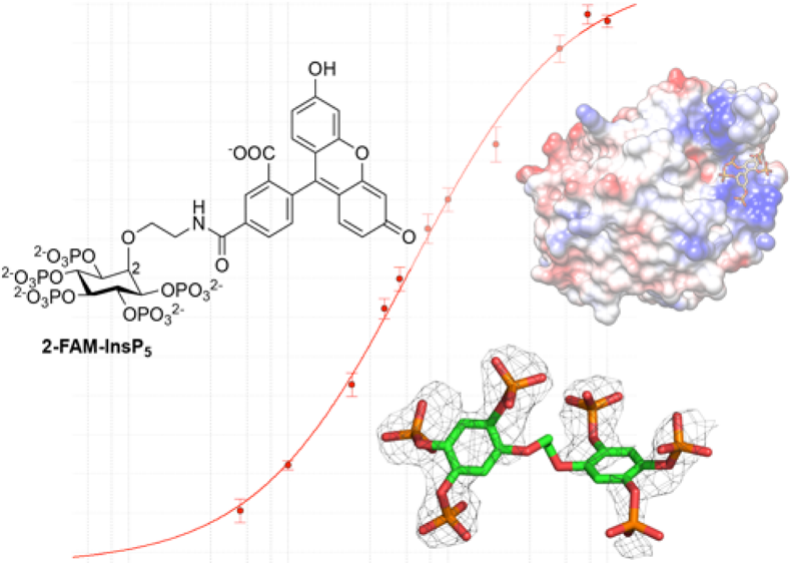

Src homology 2 domain-containing inositol phosphate phosphatase 2 (SHIP2) is one of the 10 human inositol phosphate 5-phosphatases. One of its physiological functions is dephosphorylation of phosphatidylinositol 3,4,5-trisphosphate, PtdIns(3,4,5)P_3_. It is therefore a therapeutic target for pathophysiologies dependent on PtdIns(3,4,5)P_3_ and PtdIns(3,4)P_2_. Therapeutic interventions are limited by the dearth of crystallographic data describing ligand/inhibitor binding. An active site-directed fluorescent probe facilitated screening of compound libraries for SHIP2 ligands. With two additional orthogonal assays, several ligands including galloflavin were identified as low micromolar Ki inhibitors. One ligand, an oxo-linked ethylene-bridged dimer of benzene 1,2,4-trisphosphate, was shown to be an uncompetitive inhibitor that binds to a regulatory site on the catalytic domain. We posit that binding of ligands to this site restrains L4 loop motions that are key to interdomain communications that accompany high catalytic activity with phosphoinositide substrate. This site may, therefore, be a future druggable target for medicinal chemistry.

## Introduction

Multiple mammalian enzymes share a conserved (inositol) 5-phosphatase domain, and these include INPP5A, INPP5B, INPP5E, INPP5J, OCRL, SKIP, Synaptojanin 1, Synaptojanin 2, SHIP1, and SHIP2.^1,2^ While the physiological substrates of these enzymes are not all clearly defined some, for example, SHIP1 and SHIP2, that act physiologically on PtdIns(3,4,5)P_3_ have become therapeutic targets, not least because of the restricted expression of SHIP1 in blood cell lineages.^3^ Because of this, there remains considerable interest in tools enabling identification of small molecules that influence the activity of specific enzymes.^4,5^ These small molecules may be inhibitors or activators and may also represent active site (orthosteric) or allosteric modulators. Inhibitors and/or activators have been identified for SHIP1 and SHIP2 and for OCRL and the related enzyme INPP5B.^6−15^

Irrespective of the mode of inhibition or activation of inositide- or phosphoinositide-metabolizing enzymes, the efficacy of inhibitors or activators reflects binding and subsequent catalytic processing of the bound inositide/phosphoinositide substrate. There remains an unmet need for development of assays that can distinguish between the sites of binding of substrates, substrate analogues, inhibitors, and activators whether to the active or allosteric sites.

Here, a direct fluorescence polarization (FP) methodology^16^ is shown to be suitable for characterization of phosphoinositide phosphatases such as SHIP2. The FP approach involves displacement of a fluorescent probe from the protein with a competing ligand or allosteric regulator. We note prior description of measurement of lipid phosphatase reaction products by indirect end-point competition assays employing FP probes.^12,17^ By the use of orthogonal approaches, several lead compounds are identified. Simple phosphate release and HPLC assays confirm the lead compounds to be potent inhibitors of SHIP2. The HPLC approach, itself, obviates the use of radiolabeled substrate and is able to confirm specifically inhibition of the 5-phosphatase activity of SHIP2. Finally, using a structural biology approach, we solve the structure of a SHIP2 catalytic domain in complex with a bound dimeric ligand and potent inhibitor. Binding of the ligand engages a critical residue that was recently shown to underlie allosteric control of the catalysis by C2-domain interaction with the phosphatase domain.^18^ Our study reveals a potentially druggable allosteric site on the enzyme.

## Results and Discussion

### 2-FAM-InsP_5_ Binds to SHIP2 at the Catalytic Site

**Figure 1:**
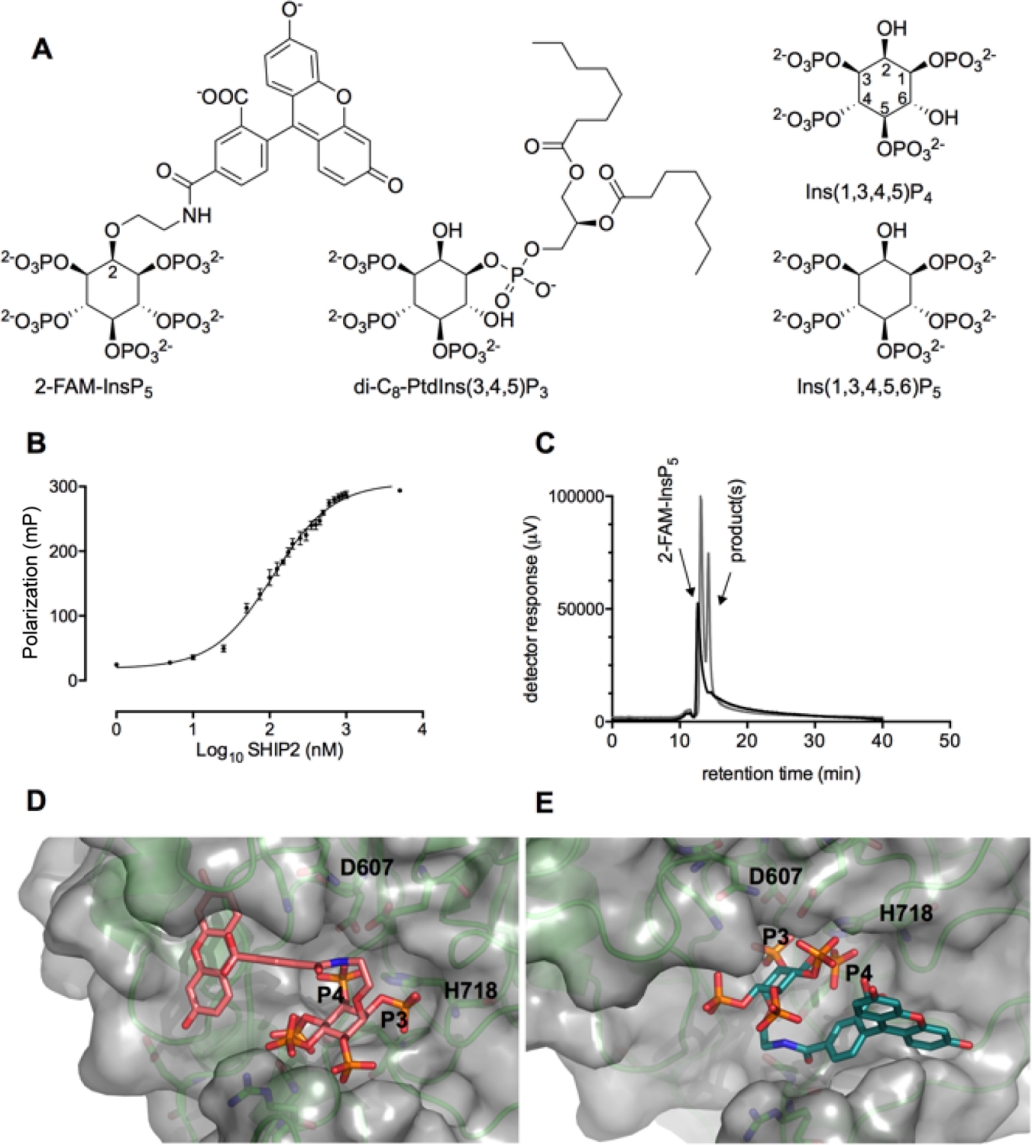
Active-site binding of 2-FAM-InsP_5_ to SHIP2cd. (A) Structures of SHIP2 substrates and 2-FAM-InsP_5_. (B) Isotherm for binding of 2-FAM-InsP_5_ to SHIP2cd. (C) Catalytic processing of 2-FAM-InsP_5_ incubated with (gray) or without (black) SHIP2cd; different amounts of sample were injected. (D, E), Docking simulations of 2-FAM-InsP_5_ binding to apo SHIP2cd reveal binding modes which place phosphates P3 and P4 close to the catalytic residues H718 and D607.

2-FAM-InsP_5_ has been shown to be a promising active-site probe of both inositol pentakisphosphate 2-kinase^19^ and human SHIP2.^16^Figure 1A compares the structure of 2-FAM-InsP_5_ with the physiological substrate PtdIns(3,4,5)P_3_, its water-soluble head group, Ins(1,3,4,5)P_4_, and Ins(1,3,4,5,6)P_5_. For human SHIP2, 2-FAM-InsP_5_ enabled identification of catalytic activity against benzene phosphate surrogates of inositide substrates.^16^ Here, we explore the binding of 2-FAM-InsP_5_ to the catalytic domain of human SHIP2 in greater detail, hereafter SHIP2cd. This was determined by measurement of probe polarization as a function of protein concentration, yielding an EC_50_ of 121 nM (Figure 1B). Products of prolonged incubation of 2-FAM-InsP_5_ at the substrate level (50 μM) were analyzed by anion-exchange HPLC with fluorescence detection at the fluorescein emission wavelength. 2-FAM-InsP_5_ appeared as a single peak by HPLC and was converted in an enzyme-dependent manner into products resolvable by HPLC (Figure 1C), confirming that, under prolonged incubation, it is a substrate. In the absence of potential product(s) for use as chromatographic standards from chemical synthesis, we are unable to identify the product of enzyme action.

In an attempt to confirm active-site binding of 2-FAM-InsP_5_, we undertook crystallography and solved an apo crystal structure of SHIP2cd (PDB 6SRR, Table S2), but despite extensive attempts at cocrystallization or soaking with 2-FAM-InsP_5_, we did not obtain complexes with this ligand. Instead, we turned to an *in silico* docking approach using the apo structure (PDB 6SRR) as the receptor with 2-FAM-InsP_5_ as a flexible ligand. The binding modes all placed the inositol phosphate ring into the active-site region of SHIP2cd and were studied for their positioning of phosphates close to the active-site catalytic residues. In the absence of an inositide substrate-liganded structure for SHIP2, detailed structural study of the close family member INPP5B^20^ has identified a number of residues conserved among the 5-phosphatase family including (for SHIP2) K541, S654, Y661, R682, and N684 that likely coordinate phosphate and hydroxyl substituents of inositide substrates. The lowest energy-binding pose predicted (Figure 1D) places the P4-phosphate of 2-FAM-InsP_5_ close to catalytic residues H718 and D607 and the FAM moiety contacting K541, projected between the L2 loop and the loop near S564. These reflect the predicted contacts of the phosphates and lipid chains of di-C_8_-PtdIns(3,4,5)P_3_, respectively (PDB 5OKM(18)). More than half of the binding modes share a similar position for the FAM moiety. A further low energy pose lying only 1.1 kcal mol^–1^ above this predicted 2-FAM-InsP_5_ to be bound in a very similar position with regards to the inositol phosphate ring, where P4 is close to H718 and P3 is close to D607 (Figure 1E). However, this binding mode placed the FAM moiety in the opposite orientation to that described (Figure 1D) and into the channel created by a loop, L4, comprising residues 674–684.

**Figure 2:**
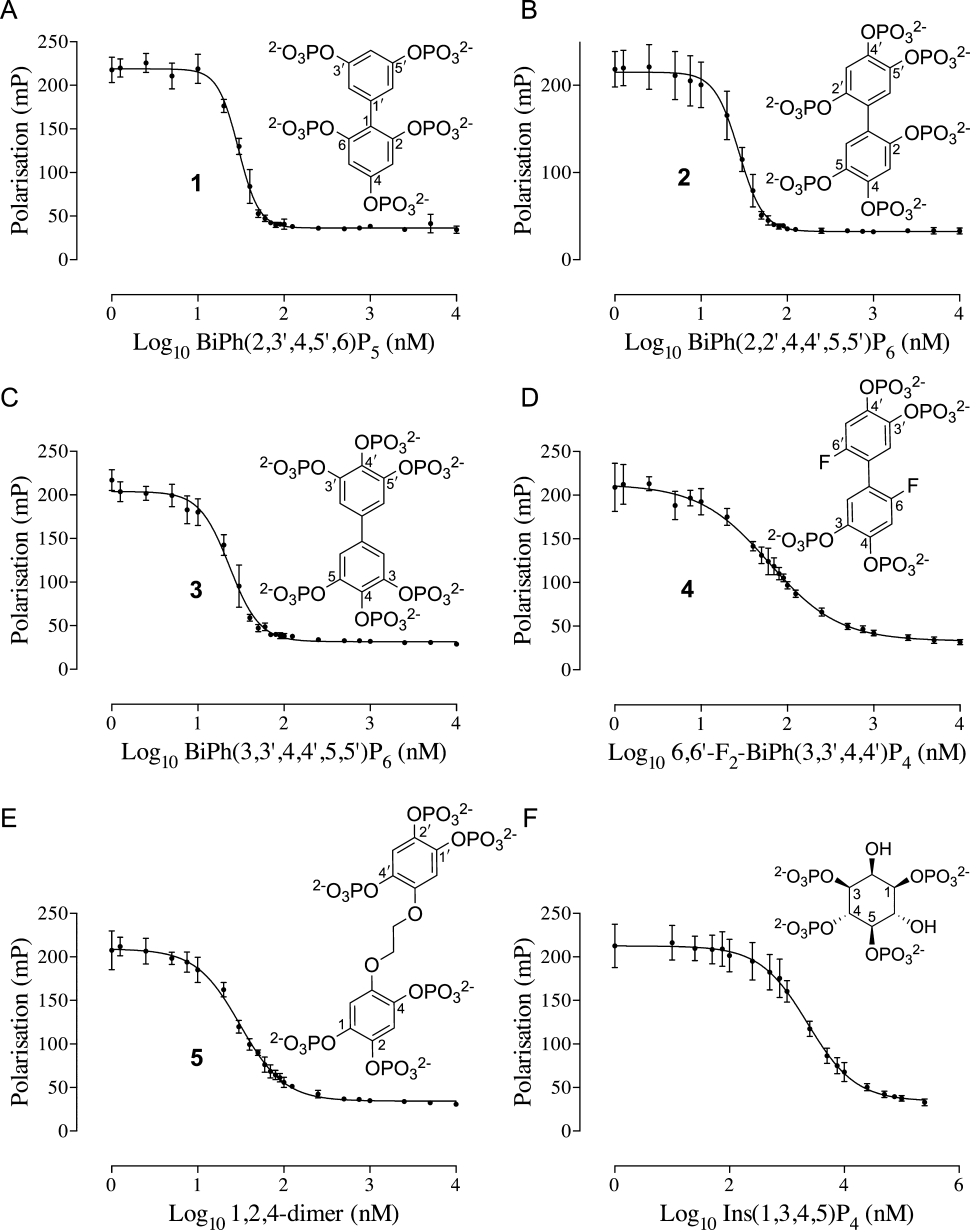
Displacement of 2-FAM-InsP_5_ from SHIP2. (A) BiPh(2,3′,4,5′,6)P_5_ (**1**), (B) BiPh(2,2′,4,4′,5,5′)P_6_ (**2**), (C) BiPh(3,3′,4,4′,5,5′)P_6_ (**3**), (D) 6,6′-F_2_-BiPh(3,3′,4,4′)P_4_ (**4**), (E) 1,2,4-dimer (**5**), and (F) Ins(1,3,4,5)P_4_. Means with SD.

These docking results, together with polarization and HPLC data suggest that 2-FAM-InsP_5_ has ample room to bind in the active site and, further to this, can place a phosphate close enough to the active-site catalytic residues H718 and D607 to allow hydrolysis to occur. It is not clear whether this is a single discrete phosphate or whether the enzyme is capable of removing several phosphates sequentially. While the unavailability of potential hydroxy-substituted products limits further analysis, a wider family of FAM/FITC-derivatized InsPs was tested as active-site probes (Table S1), confirming preferential binding of the most highly phosphorylated ligands and indicative perhaps of different binding orientations. The very slow metabolism of 2-FAM-InsP_5_ that we observed is consistent with the lack of use by SHIP2 of Ins(1,3,4,5,6)P_5_ as the substrate; Ins(1,2,3,4,5)P_5_ is the best substrate.^21^ Metabolism does however identify 2-FAM-InsP_5_ as an active-site ligand. This important property allows use of 2-FAM-InsP_5_ in active-site targeted screens. Consequently, we tested the ability of Ins(1,3,4,5)P_4_, a commonly assayed substrate in screens for inhibition of phosphatase activity,^10^ to displace 2-FAM-InsP_5_ and obtained an IC_50_ of 2300 nM (Figure 2, Table 1).

**Table 1:** Binding and Inhibition Parameters for Ligand-SHIP2 Interaction Measured by Displacement of 2-FAM-InsP_5_ (Polarization), Inhibition of Phosphate Release from Ins(1,3,4,5)P_4_, or Inhibition of Ins(1,3,4)P_3_ Production from Ins(1,3,4,5)P_4_^a^

	Δpolarization IC_50_ (95% confidence values)	phosphate release IC_50_ (95% confidence values)	conversion to Ins(1,3,4)P_3_ IC_50_ (95% confidence values)
BiPh(2,3′,4,5′,6)P_5_ (**1**)	29 nM (28–31)	3.6 μM (2.6–4.8)	3.4 μM (2.6–4.5)
BiPh(2,2′,4,4′,5,5′)P_6_ (**2**)	27 nM (26–29)	1.6 μM (1.2–2.0)	0.9 μM (0.3–2.3)
BiPh(3,3′,4,4′,5,5′)P_6_ (**3**)	23 nM (21–25)	1.0 μM (0.8–1.1)	0.7 μM (0.4–1.4)
6,6′-F_2_-BiPh(3,3′,4,4′)P_6_ (**4**)	61 nM (56–67)	19.1 μM (0.4–967)	2.5 μM (0.3–1950)
1,2,4-dimer (**5**)	31 nM (29–33)	11.0 μM (6.05–20.0)	2.7 μM (0.2–30.8)
Ins(1,3,4,5)P_4_	2300 nM (2120–2510)	N/A	N/A
purpurogallin (**10**)	5.5 μM (4.7–6.4)	89.0 μM (2.14–3696)	7.3 μM (4.5–12.0)
galloflavin (**11**)	not fitted	1.8 μM (1.04 to 3.16)	2.6 μM (2.4–2.8)
estramustine phosphate (**12**)	35.5 μM (32.4–38.9)	not fitted	41.7 μM (18.0–96.3)
5,6,7,8,4′-pentahydroxyflavone (**13**)	no displacement	6.0 μM (3.4–10.7)	not fitted
AS1949490 (**7**)	interference	1.5 μM (0.3–7.6)	not fitted

a For these experiments, EC_50_ for 2-FAM-InsP_5_ binding = 121 nM with displacement measured with polarization set at *ca.* 215 mP in the absence of the displacing ligand.

We additionally included a number of compounds that some of us have described previously as inhibitors of SHIP2,^22^ antagonists of IP_3_R,^23^ and stabilizing ligands of 5-phosphatases.^20^ Among these inositol phosphate surrogates, biphenyl 2,3′,4,5′,6-pentakisphosphate [BiPh(2,3′,4,5′,6)P_5_]^24^ (**1**) is the only ligand (substrate, analogue, or inhibitor) solved in a crystal structure bound to SHIP2 so far.^22^ Here, BiPh(2,3′,4,5′,6)P_5_ (**1**) displaced the probe with an IC_50_ of 29 nM (Figure 2A), very similar to values obtained with the closely related molecules biphenyl 2,2′,4,4′,5,5′-hexakisphosphate [BiPh(2,2′,4,4′,5,5′)P_6_] (**2**), 27 nM, and biphenyl 3,3′,4,4′,5,5′-hexakisphosphate [BiPh(3,3′,4,4′,5,5′)P_6_] (**3**), 23 nM, (Figure 2B,C). We also introduce and evaluate here the novel related analogue 6,6′-difluoro biphenyl 3,3′,4,4′-tetrakisphosphate [6,6′-F_2_-BiPh(3,3′,4,4′)P_4_] (**4**), in which one of the phosphates (on each ring) has been replaced by a fluorine; this compound gave an IC_50_ of 61 nM (Figure 2D). The related compound, 5,5′-ethane-1,2-diylbis(oxy)*bis*(benzene-1,2,4-trisphosphate)^20^ (**5**) (hereafter named 1,2,4-dimer), is a bridged dimer of the surrogate ligand of IP_3_Rs, benzene 1,2,4-trisphosphate. It bears lipid headgroup mimics separated by a spacer rather than directly through a biphenyl-type structure. It gave an IC_50_ of 31 nM (Figure 2E).

The similarities of IC_50_ for all except the difluoro compound shows that the additional phosphate(s), missing in the difluoro compound, contributes to tighter binding. Indeed, the physiological substrate, PtdIns(3,4,5)P_3_ also provides three monoester phosphates on its single ring. All the biphenyl and related compounds were more potent displacers of 2-FAM-InsP_5_ than Ins(1,3,4,5)P_4_ (Figure 2F).

The biphenyl-type phospholipid headgroup surrogates are clearly not druglike in nature. The successful binding of 2-FAM-InsP_5_ to SHIP2 and its displacement by substrates and substrate analogues led us therefore to screen the NCI Diversity set II, Developmental Therapeutics Program NCI/NIH, and the NCI Approved Oncology Drug (AOD) Set before developing assays in 384-well microtiter plates. Previously, for inositol pentakisphosphate 2-kinase, we were successful in identifying nonphosphate-containing ligands that could substitute in polyphosphate binding sites.^19^ Here, compounds were tested as singletons at 10 μM concentration in 0.1% DMSO for their ability to displace 2-FAM-InsP_5_ (5 nM) from 100 nM protein in a 100 μL volume.^19^

**Figure 3:**
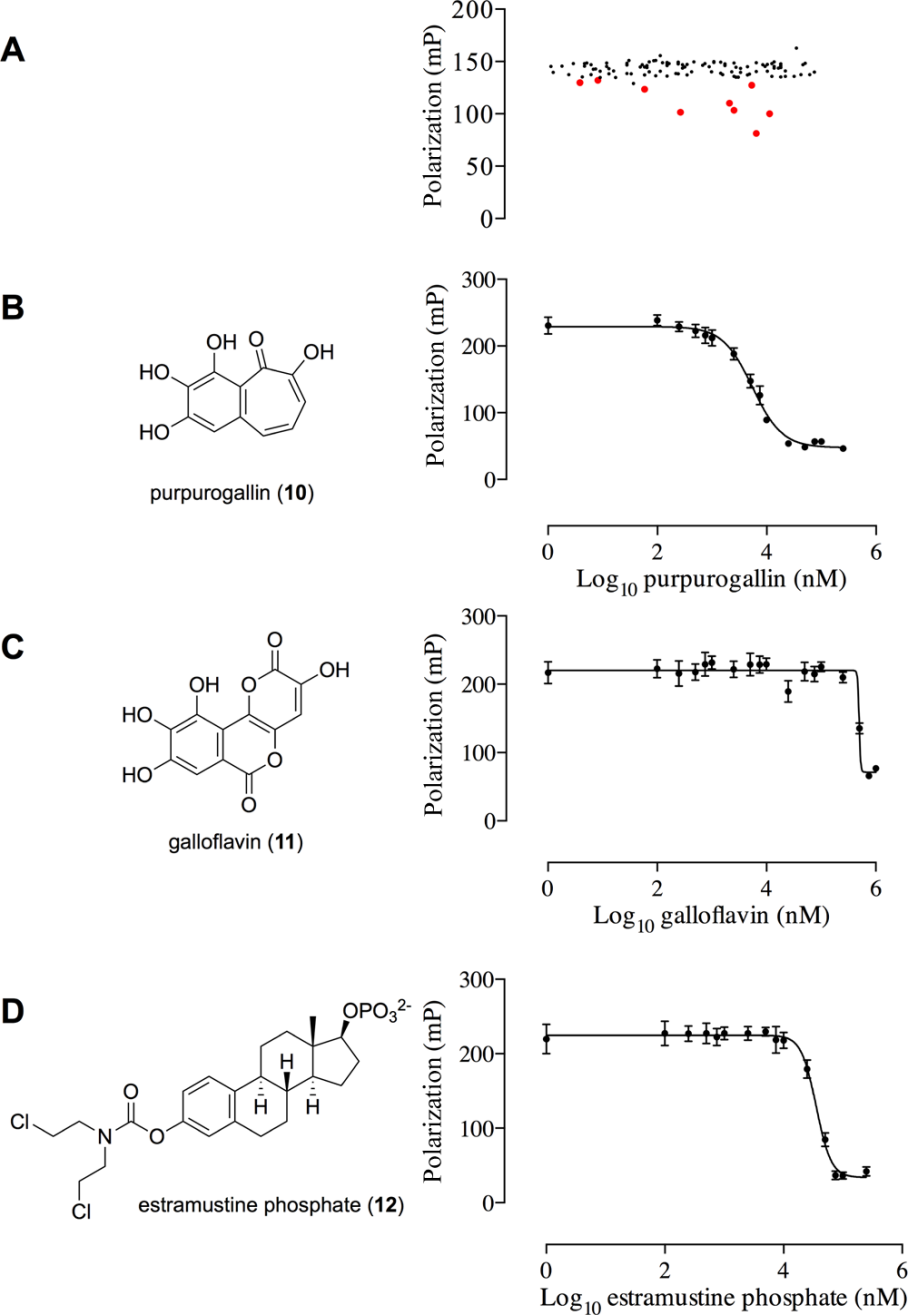
Inhibition of 2-FAM-InsP_5_ binding to SHIP2cd. (A) Displacement by AOD compounds at 10 μM; compounds in red were taken forward for further analysis. Similar data were obtained for the NCI Diversity set II. Of these, dose response of inhibition is shown (B) for purpurogallin (**10**), (C) for galloflavin, (**11**), and (D) for estramustine phosphate (**12**). Mean values with SD.

We also tested 3α-aminocholestane (**6**), a selective inhibitor of SHIP1, and AS1949490 (**7**), a selective inhibitor of SHIP2^6,7,10^ (Figure S1). 3α-Aminocholestane (**6**) increased polarization of the probe but did so in the absence of protein. We attribute this to aggregation of 2-FAM-InsP_5_ into compound micelles. AS1949490 also interfered in the FP assay. From the AOD set, both bosutinib (**8**) and crizotinib (**9**) (Figure S1), the latter with a reported IC_50_ of 5.5 μM for inhibition of SHIP2,^13^ similarly increased polarization of the probe. Compounds that reduced polarization below 100 mP were taken forward for dose–response analysis in the range 1 nM–100 μM in 384-well plates. Diversity Set II yielded purpurogallin (**10**) and galloflavin (**11**) as displacing ligands with IC_50_s of 5.5 μM and approximately 500 μM (Figure 3B,C), while the AOD set yielded estramustine phosphate (**12**) with IC_50_ 35.5 μM (Figure 3D). Mindful of the possibility of identification of “false-positives” by virtue of undefined interactions in the polarization assay, we constructed two secondary screens.

**Figure 4:**
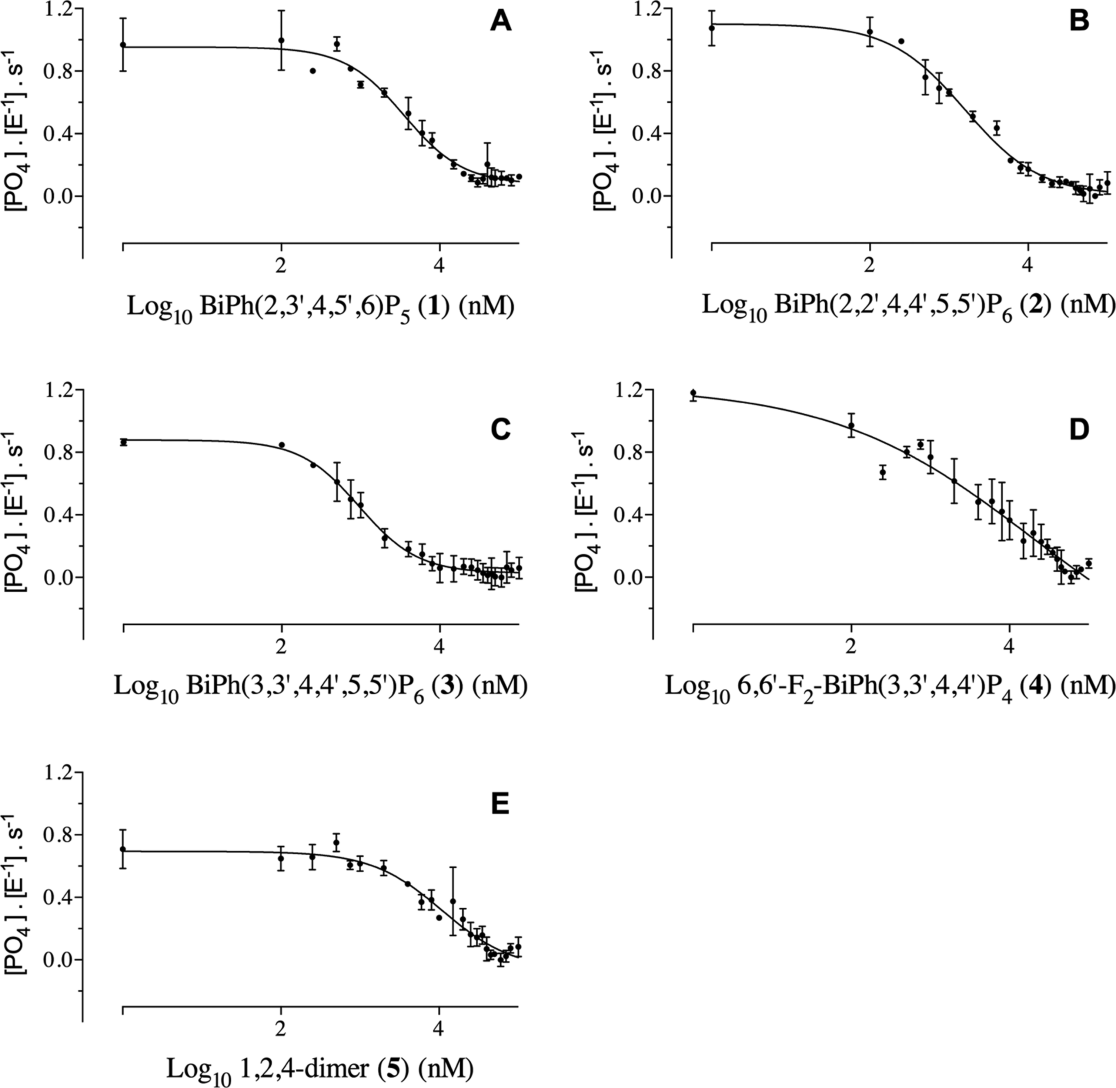
Inhibition of SHIP2cd enzyme activity. Phosphate release from Ins(1,3,4,5)P_4_ is inhibited by (A) BiPh(2,3′,4,5′,6)P_5_ (**1**), (B) BiPh(2,2′,4,4′,5,5′)P_6_ (**2**), (C) BiPh(3,3′,4,4′,5,5′)P_6_ (**3**), (D) 6,6′-F_2_-BiPh(3,3′,4,4′)P_4_ (**4**), and (E) 1,2,4-Dimer (**5**). Mean values with SD.

We performed assays of the ability of purpurogallin (**10**), galloflavin (**11**), estramustine phosphate (**12**), AS1949490 (**7**), and 5,6,7,8,4′-pentahydroxyflavone (**13**) to inhibit the Ins(1,3,4,5)P_4_ phosphatase activity of SHIP2, measured as release of phosphate. Galloflavin (**11**), 5,6,7,8,4′-pentahydroxyflavone (**13**), and AS1949490 (**7**) gave IC_50_ < 10 μM (1.8, 6.0, and 1.5 μM, respectively) (Table 1). The value for AS1949490 (**7**) is similar to the 0.44 ± 0.19 μM Ki for inhibition of phosphate release from Ins(1,3,4,5)P_4_ reported for this compound^10^ and the identical value 0.44 ± 0.07 μM reported for the related compound AS1938909.^14^ Neither compound has been crystallized with SHIP2, but others have docked AS1938909 with modeled SHIP2 (PDB 4A9C) and report poses of AS1938909 and crizotinib (**9**) in the active site.^13^ The biphenyl phosphates and related compounds were comparable inhibitors to galloflavin (**11**) and AS1949490 (**7**), yielding IC_50_s for BiPh(2,3′,4,5′,6)P_5_ (**1**), 3.6 μM; BiPh(2,2′,4,4′,5,5′)P_6_ (**2**), 1.6 μM; BiPh(3,3′,4,4′,5,5′)P_6_ (**3**), 1.0 μM; 6,6′-F_2_-BiPh(3,3′,4,4′)P_4_ (**4**), 19.1 μM; and 1,2,4-dimer (**5**), 11.0 μM (Figure 4).

**Figure 5:**
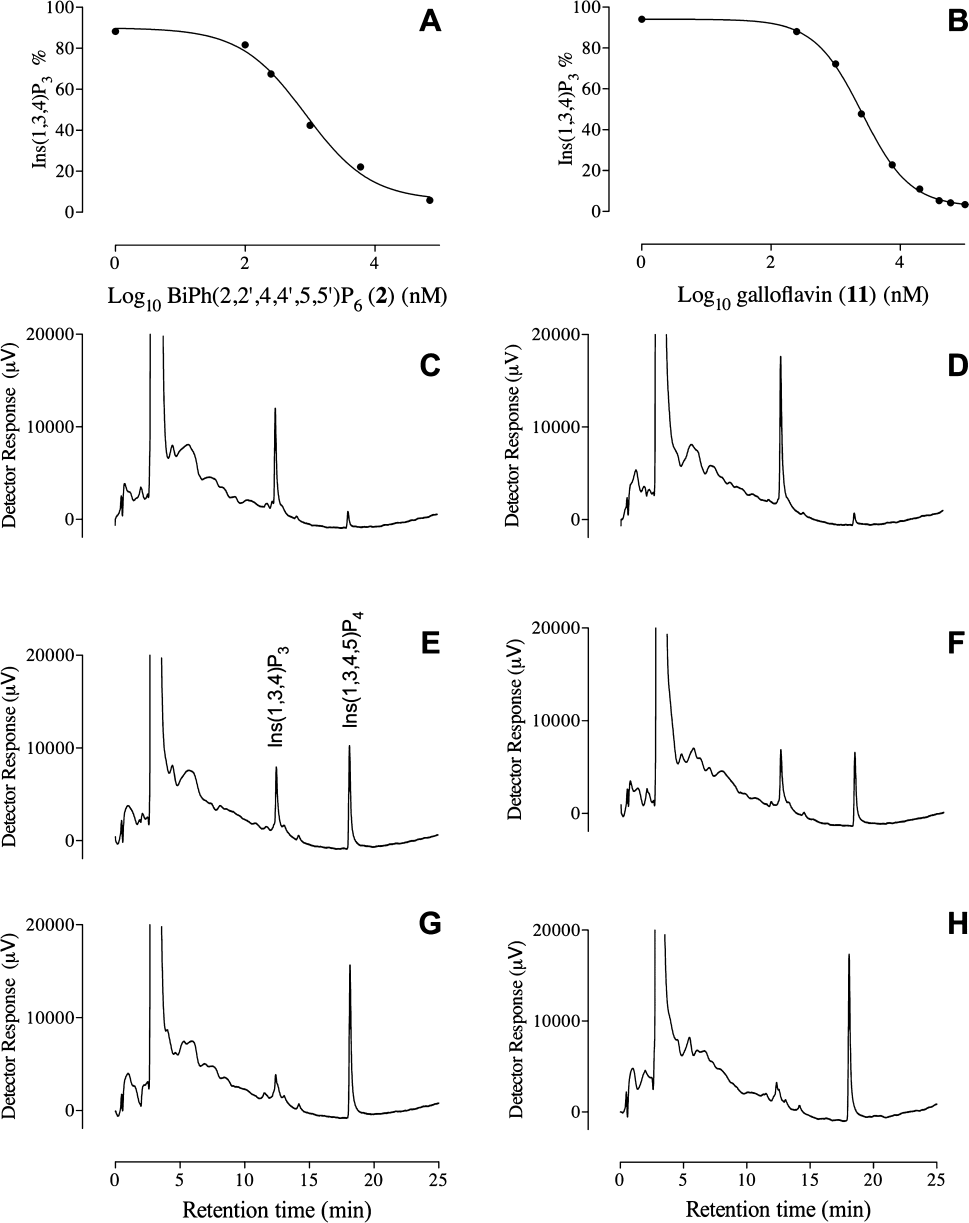
Postcolumn metal-complexation HPLC identifies inhibitors of SHIP2 5-phosphatase activity. SHIP2cd-mediated 5-dephosphorylation of Ins(1,3,4,5)P_4_ is inhibited by (A) BiPh(2,2′,4,4′,5,5′)P_6_ (**2**), (B) galloflavin (**11**). HPLC separation of substrates and products are shown at progressively increasing concentrations of inhibitor (C,E,G) BiPh(2,2′,4,4′,5,5′)P_6_ (**2**) and (D,F,H) galloflavin (**11**).

Nevertheless, we sought alternative validation of the inhibitors identified in the prior two screens. Because neither the 2-FAM-InsP_5_ FP nor phosphate-release assays testify to the specificity of phosphatase attack on the Ins(1,3,4,5)P_4_ substrate, we further used postcolumn metal-complexation HPLC to confirm the efficacy of inhibitors identified above. HPLC confirmed SHIP2-catalyzed production of Ins(1,3,4)P_3_ (Figure S2), inhibited by BiPh(2,3′,4,5′,6)P_5_ (**1**), BiPh(2,2′,4,4′,5,5′)P_6_ (**2**), BiPh(3,3′,4,4′,5,5′)P_6_ (**3**), 6,6′-F_2_-BiPh(3,3′,4,4′)P_4_ (**4**), and 1,2,4-dimer (**5**) (Table 1). Similarly, galloflavin (**11**), estramustine phosphate (**12**), and purpurogallin (**10**) yielded IC_50_s for inhibition of Ins(1,3,4)P_3_ production of 2.6, 41.7 and 7.3 μM, respectively (Table 1). Example traces from this HPLC assay are shown for BiPh(2,2′,4,4′,5,5′)P_6_ (**2**) and galloflavin (**11**) (Figure 5).

The fitted curves for the complete set of biphenyl compounds and 1,2,4-dimer (**5**) are shown (Figure S3) and for other compounds (Figure S4). AS1949490 (**7**), 5,6,7,8,4′-pentahydroxyflavone (**13**), and valrubicin (**14**) were also tested (the structures of the drug-like compounds are shown in Figure S1); valrubicin (**14**) appeared to be effective, but at the highest concentrations, it showed deviation from monotonic behavior, which we think is likely caused by insolubility of the compound in the assay at the higher concentrations (Figure S4). Overall, these assays show that galloflavin (**11**) and 5,6,7,8,4′-pentahydroxyflavone (**13**) are, by well-used phosphate release assays, of similar effect to AS1949490 (**7**). Like AS1949490 (**7**), they do not modify the nature of the inositol phosphate product of SHIP2 action. Moreover, we show that metal-complexation HPLC is a powerful test of inhibition of 5-phosphatase activity.

**Figure 6:**
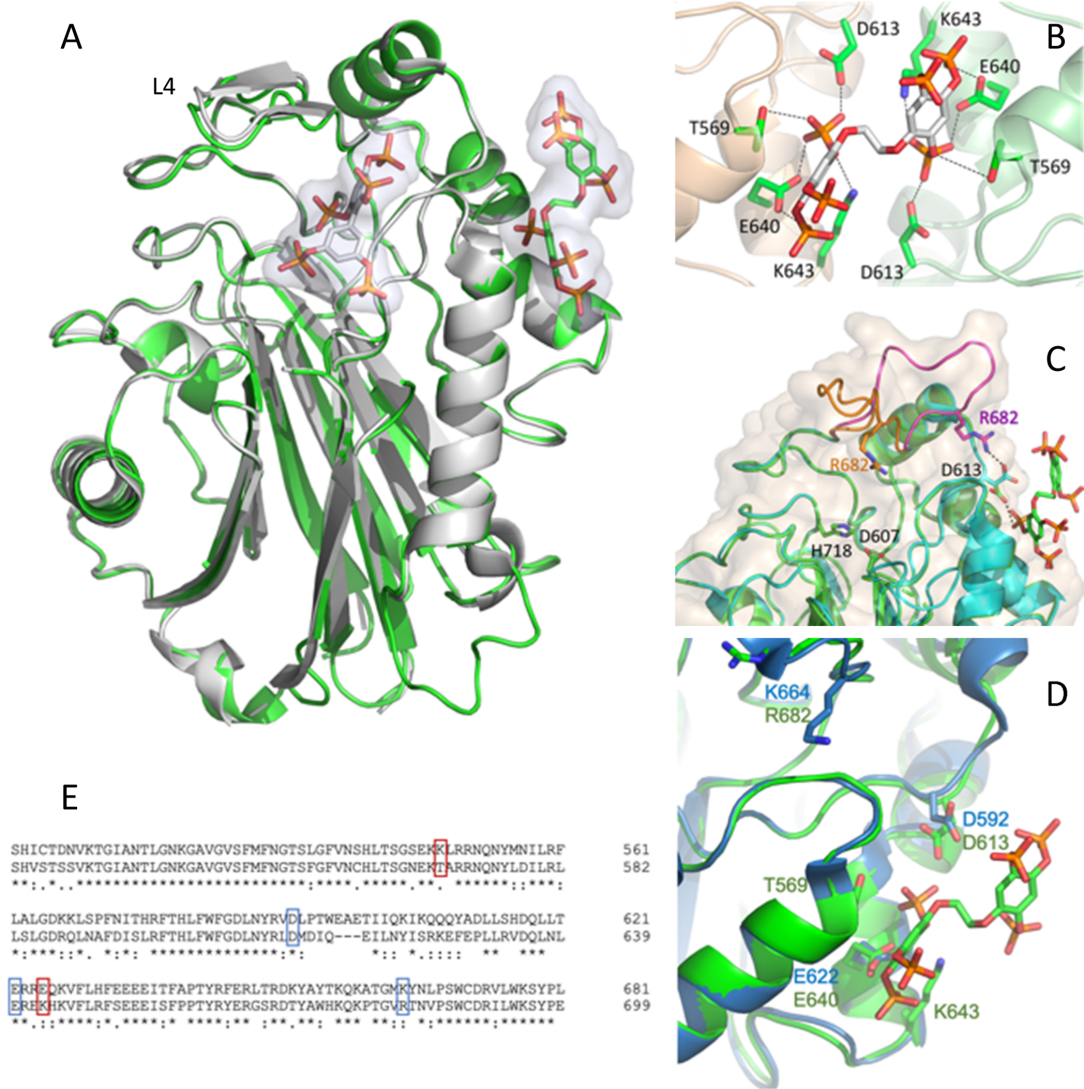
An allosteric site on SHIP2cd. (A) Overlay of SHIP2:1,2,4-dimer (**5**) (PDB 6SQU, green) and SHIP2: BiPh(2,3′,4,5′,6)P_5_ (**1**) (PDB 4A9C, gray). Loop L4^18^ is indicated (see text). (B) SHIP2 interactions with 1,2,4-dimer (**5**). Viewed approximately along the pseudo-twofold axis relating protein monomers in the asymmetric unit, individual protein monomers are shown in wheat (monomer B) and green (monomer A). (C) Detail from the superposition of structures PDB 6SQU (green cartoon; orange L4 loop in L4-in conformation) and 5OKM (cyan cartoon; magenta L4 loop in L4-out conformation). Catalytic residues D607 and H718 are labeled, as is R682 on the L4 loop. Note that D613 adopts two conformations to interact with either R682 (L4-out conformation) or with the inhibitor 1,2,4-dimer (**5**) (L4-in conformation). Hydrogen bonds are shown as dashed lines. Molecular surfaces are shown in wheat. (D) Overlay of SHIP2:1,2,4-dimer (**5**) (PDB 6SQU, green) and human SHIP1 (PDB 6IBD, blue) in the region of the 1,2,4-dimer binding site. Residues in contact with 1,2,4-dimer in PDB 6SQU are shown in a stick format and labeled, as are the corresponding residues in PDB 6IBD. Residue R682 in SHIP2 and its partner in SHIP1 are also shown. (E) Clustalw alignment of human SHIP1 (PDB 6IBD), top sequence, and human SHIP2 (PDB 6SQU), lower sequence. Residues contacting 1,2,4-dimer in PDB 6SQU are highlighted; conserved or not (blue or red boxes).

Crystal structures have been solved for a number of truncated SHIP2 proteins.^18,22,25^ However, the only structure solved with a ligand, other than buffer ions, is that of the catalytic domain in complex with BiPh(2,3′,4,5′,6)P_5_ (**1**) (PDB 4A9C(22)). Structures of related 5-phosphatases have been solved in complex with few ligands and only with the catalytic domain. Among these ligands, benzene 1,2,4,5-tetrakisphosphate [Bz(1,2,4,5)P_4_] (**15**) and BiPh(3,3′,4,4′,5,5′)P_6_ (**3**) yielded complexes.^22^ Our attempts at cocrystallization of SHIP2cd with the displacing or inhibitory ligands shown in Figures 1–5 and S1–S4 were without success. Nevertheless, we were able to solve the structure of the apoenzyme (PDB, 6SRR), and in crystal soaks with 1,2,4-dimer (**5**), we obtained a structure of the complex at 2.27 Å resolution (PDB, 6SQU) (Figure 6 and Figure S5, Table S2). The crystal structure of the 1,2,4-dimer-SHIP2cd complex comprises two monomers of SHIP2 and a single copy of the inhibitor in the asymmetric unit. The space group (P2_1_) and cell parameters are essentially isomorphous with those described previously for the BiPh(2,3′,4,5′,6)P_5_–SHIP2cd complex (PDB, 4A9C(22)) and corresponding apo structure (PDB, 3NR8(25)). The most striking facet of our structure of the complex (PDB, 6SQU) is the binding of the ligand in a shallow pocket distal to the active site (Figure 6A). Superposition of PDB 4A9C and PDB 6SQU identifies the binding site for 1,2,4-dimer (**5**) ligand around 15 Å distant from that observed for BiPh(2,3′,4,5′,6)P_5_ (**1**)^22^ (Figure 6A). With an rmsd of 0.25 Å (247 Cα atoms) between the two inhibitor-bound structures, ligand binding appears not to promote major differential conformational changes.

The detailed studies of Le Coq on phosphatase-C2-domain protein (PDB 5OKM(18)) have identified how motions of the L4 loop, residues 674–684, contribute to enhanced catalysis of inositide and phosphoinositide substrates. In particular, they show that the L4 loop can, in addition to the “closed” (L4-in) over the active-site pose observed of the phosphatase domain (PDB, 4A9C(22)), take alternative open (L4-out) or intermediate poses. The L4 loop harbours an arginine (R682) that contacts either or both of a pair of aspartic acid residues (D613/D615) that are adjacent to one end of the α5 helix in the L4-out conformation. The α5 helix is one of three (α5-7) that, at the other end, are involved in a network of interactions *via* the phosphatase domain residue R649 with the C2 domain. Thus, the L4-out conformation is stabilized by a relay of interactions with the C2 domain that offers an explanation of the significant activation of enzyme for the lipid substrate afforded by this domain.^18^

While the distal binding of 1,2,4-dimer (**5**) does not significantly modify the L4 closed (L4-in) conformation from that seen in the complex with BiPh(2,3′,4,5′,6)P_5_ (**1**) (Figure 6A), the compound is a potent displacer of 2-FAM-InsP_5_ (Figure 2) and a potent inhibitor of 5-phosphatase activity against Ins(1,3,4,5)P_4_ (Table 1 and Figure S3). The 1,2,4-dimer (**5**) does not occupy the positions of the crystallographically resolved ligands or of the predicted bound states of docked ligands in catalytic domain or multidomain structures.^18^ Indeed, the 1,2,4-dimer (**5**) ligand fails to directly contact the L4 loop (Figure 6). Remarkably, we observe that phosphate P4 of 1,2,4-dimer (**5**) interacts with D613 (Figure 6B,C). This, we suggest, precludes interaction of this residue with R682 and adoption of the L4-out conformation that, in its transient motions, accompanies optimal catalysis.^18^ Two of the four residues coordinating 1,2,4-dimer, D613 and E640, are conserved in SHIP1 (D592 and E622, respectively). The L4-loop residue R682 is also conservatively replaced with K664 (Figure 6D,E). Given the conservation of the L-4 loop in SHIP1 and SHIP2, these observations suggest that a common mechanism of inhibition could be shared between SHIP1 and SHIP2.

A recent study of the closely related enzyme SHIP1 has proposed, on the basis of docking and site-directed mutagenesis, that allosteric regulators of the Pelorol family, ZPR-MN100 (formerly known as AQX-MN100) and ZPR-151, bind in the interface between the C2 and catalytic domain of SHIP1.^26^ The binding site for these ligands is proposed to include a lysine residue (K681 in PDB entry 6DLG) that is approximately 23.7 Å distant from D613 coordinated by 1,2,4-dimer (6SQU) (Figure S6). Thus, while the two allosteric agents ZPR-MN100 and 1,2,4-dimer occupy distinct sites, they both point to interdomain (C2-catalytic) communication as a target for therapeutic manipulation.

**Figure 7:**
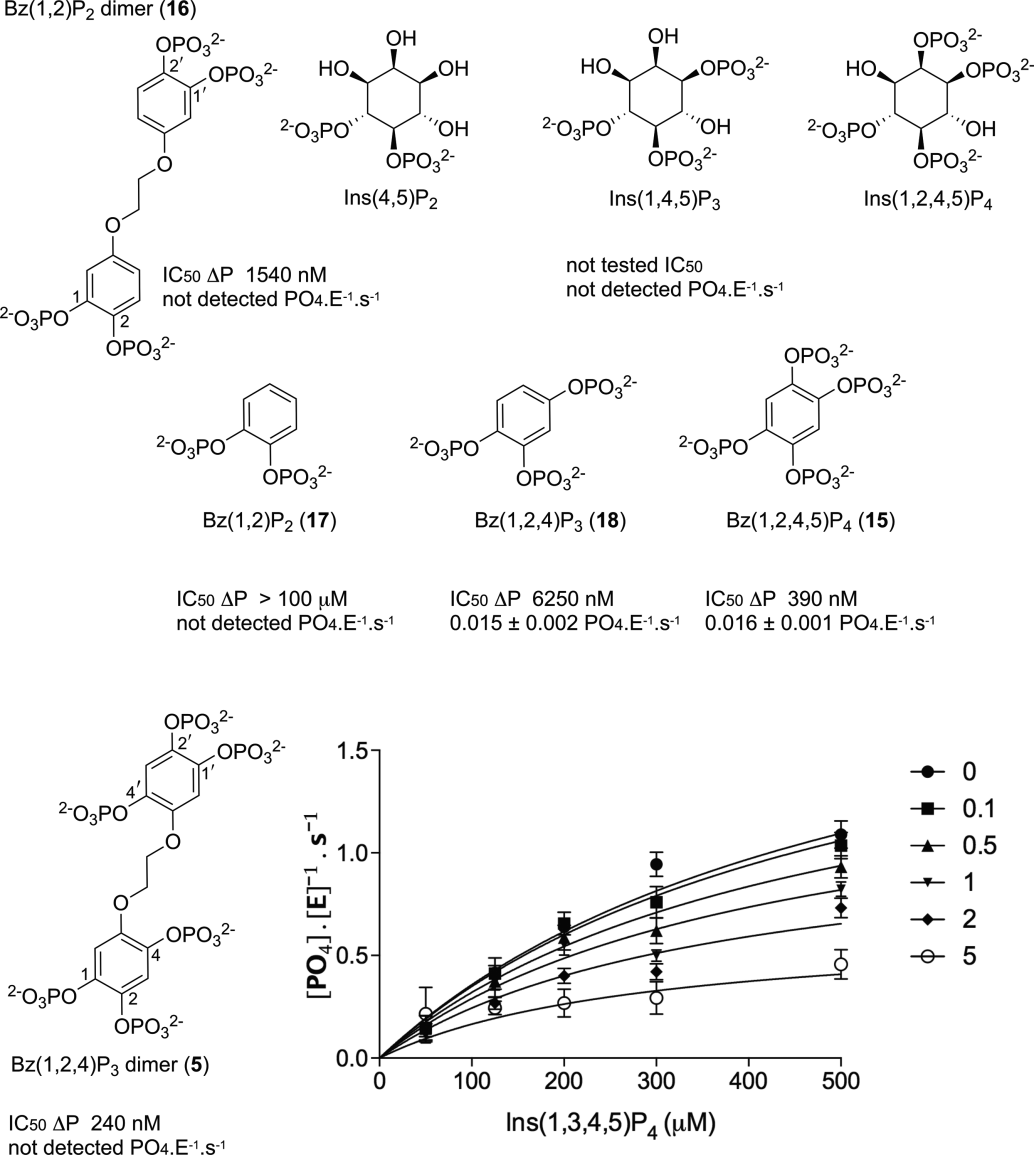
Substrate preference of SHIP2 for benzene phosphates and related dimers. Benzene phosphates, related molecules, and inositol phosphate analogues of benzene phosphates are shown; Ins(4,5)P_2_, Ins(1,4,5)P_3_, and Ins(1,2,4,5)P_4_ are not substrates.^21^ Catalytic activity toward compounds is reported as phosphate release, measured at 100 μM substrate and 1 or 10 μM enzyme. The figure additionally shows IC_50_ values for displacement of 2-FAM-InsP_5_. For these experiments, IC_50_ for 2-FAM-InsP_5_ binding = 820 nM, with displacement measured with the polarization set at 125 mP in the absence of the displacing ligand. The bottom-right panel shows inhibition of SHIP2 activity against the Ins(1,3,4,5)P_4_ substrate by 1,2,4-dimer in the range 0.1–5 μM at an enzyme concentration of 100 nM. The data were fitted by nonlinear least squares regression to a mixed inhibition model in GraphPad v6. Values for enzymatic activity are means and standard deviation of four measurements.

To explore the structure-activity relationship of the 1,2,4-dimer-SHIP2cd interaction, we extended our analysis to close analogues of the 1,2,4-dimer (**5**). These include 4,4′-ethane-1,2-diyl*bis*(oxy)*bis*(benzene-1,2-bisphosphate) (hereafter named 1,2-dimer) (**16**) with fewer phosphates^20^ and the respective benzene phosphate monomer units Bz(1,2)P_2_ (**17**) and Bz(1,2,4)P_3_ (**18**). Bz(1,2,4)P_3_ (**18**) was originally designed as a structural analogue of Ins(1,4,5)P_3_.^23^ We included Ins(1,4,5)P_3_ itself because in an exhaustive study of *Schizosaccharomyces pombe* synaptojanin and human SHIP2, while Ins(1,4,5)P_3_ was found to be an exemplary substrate of the former [10-fold better than Ins(1,3,4,5)P_4_], it proved not to be a substrate of SHIP2.^21^ Consistent with this, neither Ins(1,4,5)P_3_ nor either of the 1,2- (**16**) or 1,2,4-dimers (**5**) or Bz(1,2)P_2_ (**17**) were substrates (Figure 7), but consistent with our recent study,^16^ Bz(1,2,4)P_3_ (**18**) and Bz(1,2,4,5)P_4_ (**15**) were efficient substrates with an activity of *ca.* 10% of Ins(1,3,4,5)P_4_.

These data begin to dissect the structural requirements for local allosteric regulation of SHIP2cd. Thus, while among inositides, the more densely phosphorylated inositol phosphates are the strongest substrates [Ins(1,2,3,4,5)P_5_ is the strongest],^21^ so it is also true for simple benzene phosphates^16^ and, importantly, their formal biphenyl counterparts (Figure S7). That is to say, biphenyl phosphates are substrates; they bind in the active site. For the simple benzene phosphate, Bz(1,2,4)P_3_ (**18**), addition of a phosphate to the 5-position retains catalytic acceptance as does substitution of the 3-position with a hydroxyl group: 3-OH-Bz(1,2,4)P_3_ (structure not shown) and Bz(1,2,4,5)P_4_ (**15**) are both progressively dephosphorylated by SHIP2.^16^ In contrast, substitution of the 5-position of Bz(1,2,4)P_3_ (**18**) with an oxo-linked ethylene spacer in 1,2,4-dimer (**5**) renders this molecule a nonsubstrate. Similar substitution of the 4 = 5 position of Bz(1,2)P_2_ (**17**) in 1,2-dimer (**16**) also did not render catalytic acceptance. The aforementioned crystallographic studies show that uniquely among the benzene phosphates, the complete set of biphenyl phosphate and 1,2,4-dimer ligands tested in crystallographic screens, 1,2,4-dimer (**5**) is a ligand of distal (to the active site) residues including D613. This residue makes a critical contribution to the relay of interactions between the C2-domain and L4 that confer enhanced turnover of lipids by the catalytic domain.^18^ We might speculate that *O*-methyl or *O*-ethyl substitution of Bz(1,2,4)P_3_ (**18**) might render this molecule a local allosteric regulator of SHIP2cd activity, like its dimer counterpart.

In support of these experiments, we tested the efficacy of the two dimer compounds and their related benzene phosphate monomers as displacers of 2-FAM-InsP_5_ (Figure 7). The IC_50_s for these simple benzene phosphate substrates further highlight the phosphate density dependence of active-site binding: Bz(1,2)P_2_ (**17**), >100 μM; Bz(1,2,4)P_3_ (**18**), 6250 nM; and Bz(1,2,4,5)P_4_ (**15**), 390 nM. The InsP analogues of these, Ins(4,5)P_2_, Ins(1,4,5)P_3_, and Ins(1,2,4,5)P_4_ are not substrates of SHIP2,^21^ and Bz(1,2,4)P_3_ is an inhibitor of the Ins(1,4,5)P_3_ phosphatase activity of bovine adrenal microsomes.^27^ Our experiments confirmed further the potency of the dimers as SHIP2cd ligands; IC_50_ for displacement of 2-FAM-InsP_5_: 1,2-dimer (**16**), 1540 nM; 1,2,4-dimer (**5**), 240 nM (Figure 7). Overall, the 1,2-dimer (**16**) and the 1,2,4-dimer (**5**) exhibit properties quite distinct from their simple benzene phosphate homologues and distinct from more densely phosphorylated biphenyl compounds.^16,20,22^ That is to say, the dimers are not substrates (Figure 7) and the 1,2,4-dimer (**5**) is an inhibitor of catalytic activity (Table 1, Figures 4, S3) that occupies a distal (to the active site) regulatory (allosteric) site (Figures 6, S5).

To test the modality of enzyme inhibition by 1,2,4-dimer (**5**), the Ins(1,3,4,5)P_4_ substrate and inhibitor [1,2,4-dimer (**5**)] were titrated. The results fitted globally to a mixed inhibition model (Figure 7) yielded a thermodynamic cooperativity factor α of 0.36. The numerical value of this parameter is diagnostic of mixed uncompetitive inhibition; for competitive inhibition, α tends to infinity.^28^ Thus, 1,2,4-dimer (**5**) meets both the kinetic and structural criteria of an uncompetitive allosteric inhibitor of SHIP2. The Ki value determined, 4.9 μM, is within the range IC_50_ (2.7–11 μM) obtained at a fixed (250 μM) substrate concentration in Table 1. The *kcat* value obtained (2.3 s^–1^) is similar to that (1.3 s^–1^) obtained for the catalytic domain by Le Coq and coauthors.^18^ In the former study, the C2 domain provided an enhancement of catalysis that was significantly greater for di-C_8_-PtdIns(3,4,5)P_3_ than for Ins(1,3,4,5)P_4_, a result consistent with cell biology that indicates that the principal physiological substrate in a number of scenarios is most likely PtdIns(3,4,5)P_3_.^6^

Intriguingly, inspection of residual electron density maps for our refined structure of the complex with 1,2,4-dimer (**5**) with SHIP2cd (PDB 6SQU) revealed a small but significant region of contiguous density in the active site for monomer B. No comparable residual density is present in monomer A (PDB 6SQU) or in either monomer of our structure of the apocatalytic domain (PDB 6SRR). The most significant density lies close to H718, a residue which has been modeled as contacting the catalytically favoured P5 phosphate of di-C_8_-PtdIns(3,4,5)P_3_ in the phosphatase-C2 structure (PDB 5OKM(18)). We used the LigandFit approach in the Phenix software suite^29,30^ in exhaustive attempts to dock 1,2,4-dimer (**5**) to this electron density, but no solutions were found (the free R-factor rose by 1.52% for the docked solution with the highest local correlation coefficient). The CC of this solution was 0.54; a value below 0.6 usually means that the ligand is misplaced because no suitable density could be found. This result was unsurprising, given the large disparity in the size and shape of the density feature compared to that of the inhibitor. These observations further discount the possibility that the contiguous electron density observed in the active site of monomer B (PDB 6SQU) corresponds to a second molecule of 1,2,4-dimer; indeed, 1,2,4-dimer is an uncompetitive inhibitor (Figure 7).

**Figure 8:**
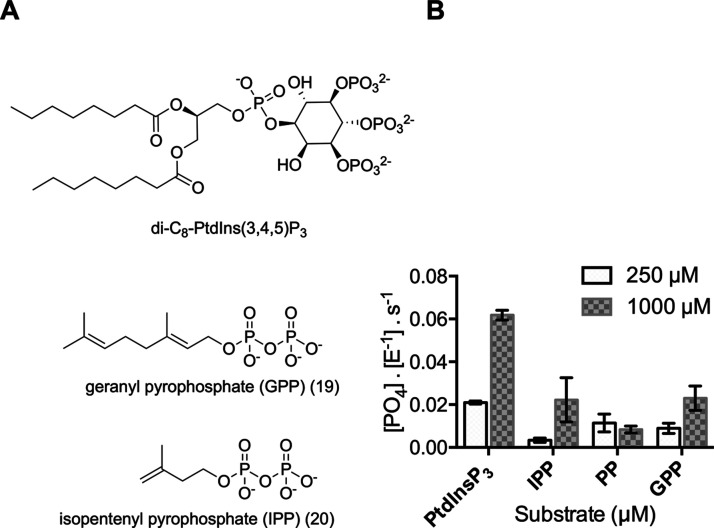
Catalytic activity of SHIP2 toward isoprenoids. (A) Structures of isoprenoids and di-C_8_-PtdIns(3,4,5)P_3_ substrates. (B) Phosphate release from di-C_8_-PtdIns(3,4,5)P_3_ geranyl pyrophosphate, GPP (**19**); isopentenyl pyrophosphate, IPP (**20**); sodium pyrophosphate, NaPP; (mean and s.d.).

As no components of the purification buffers or crystallization solution fitted this density, we next considered what the SHIP2cd protein may have contacted and potentially bound prior to its purification from the expression host. Automated ligand identification in the Phenix software suite^29,30^ carries out fitting of a library of 180 of the most frequently observed ligands in the PDB to residual electron density in a given map. We employed this procedure together with the difference electron density map from PDB 6SQU, yielding a high score (second in the ranked list; Table S3) for the isoprenoid pyrophosphate-containing compound, farnesyl diphosphate (local correlation coefficient 0.61, Z-score 1.76). Given the preference of SHIP2 for phosphoinositide substrates, pyrophosphate-containing molecules such as the FPP precursors geranyl pyrophosphate (GPP) (**19**) and isopentenyl pyrophosphate (IPP) (**20**) (Figure 8) were also considered as possible ligands. Automated fitting of these with Phenix resulted in local correlation coefficients of 0.66 and 0.52, respectively. The fit of geranyl pyrophosphate to active site residual electron density of PDB 6SQU is shown (Figure S8). We did not crystallographically refine these models as we have no independent evidence for ligand presence in the crystal. We did, however, undertake phosphate release assays, proffering GPP (**19**), IPP (**20**) and pyrophosphate as substrates for SHIP2cd. Our results demonstrate that these molecules can indeed bind in the active site and act as substrates (Figure 8A). We were unable to assay FPP due to a high-phosphate background. Phosphate release from both GPP (**19**) and IPP (**20**) at a concentration of 1 mM was found to be similar to that of di-C_8_-PtdIns(3,4,5)P_3_ at 250 μM (Figure 8A). Although there is no published evidence that GPP (**19**) is a physiological substrate, it is interesting that the SHIP2cd domain can bind and utilize pyrophosphate molecules with lipid-like methylene tails. Thus, while it is clear that GPP (**19**) is a substrate of SHIP2cd, the identity of the bound moiety (moieties) remains unresolved. While the C2-Ptase apo structure (PDB 5OKM) has limited active-site density that has been modeled to water(s),^18^ we note that GPP can be docked to the residual active site density in the human SHIP2 apo structure (PDB 3NR8), albeit in a different orientation to that shown for PDB 6SQU (Figure S8). One intriguing possibility is that a previously unrecognized substrate or product is evident in the 1,2,4-dimer (**5**)-liganded structure.

**Figure 9:**
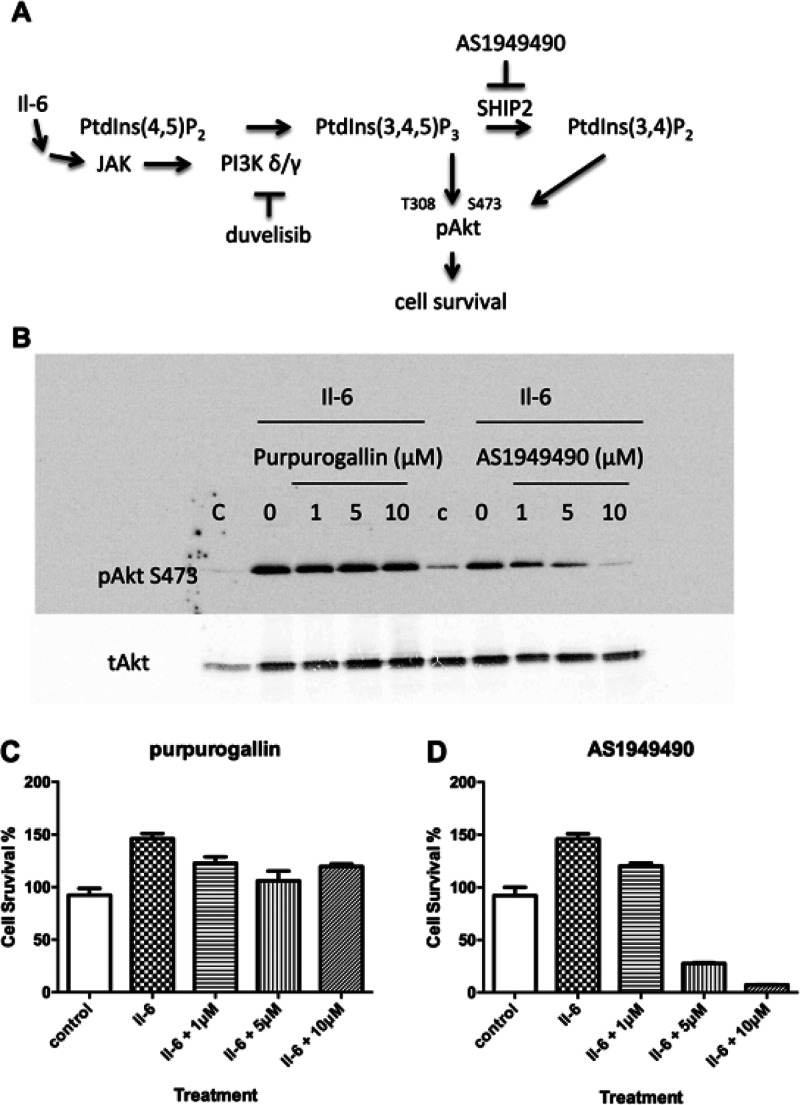
Inhibition of IL6-induced cell survival of MM1 by AS1949490 (**7**). (A) Cartoon of SHIP2 involvement in PI3K/Akt signaling in multiple myeloma cells. Il-6 activates Akt by mechanisms including specific phosphorylation of S473, the action of Il-6, including phosphorylation of S473, is blocked by inhibition of SHIP2. (B) pAKT S473 and total AKT levels in serum-starved MM1 cells (control) and cells treated for 30 min with 10ng mL^–1^ Il-6 in the presence or not of either purpurogallin (**10**) or AS1949490 (**7**). (C,D) Cell viability was measured after 24 h (mean and s.e.).

The foregoing structural and enzymological studies highlight a range of approaches and experimental observations that could enable medicinal chemistry interventions on SHIP2. PtdIns(3,4,5)P_3_ signaling in multiple myeloma has been shown to be an accessible system for study of small molecule targeting of SHIP1/2 function.^6^ We sought to test the efficacy of the putative SHIP2 inhibitors identified above in a multiple myeloma context. Interleukin 6 (Il-6) is a peptide growth factor that supports cell survival of multiple myeloma cells which express multiple PI3K isoforms constitutively.^31^ Il-6-treatment of multiple myeloma cells and cell lines activates Akt by phosphorylation of S473, and this can be blocked by inhibition of PI3Kδ/γ with the dual PI3K PI3Kδ/γ inhibitor duvesilib or by lentiviral knockdown of combined PI3Kδ/γ.^32^ A cartoon of the pathway is shown in Figure 9A.

We treated serum-starved MM1 cells (a multiple myeloma cell line) with Il-6, as a positive control, and with the inhibitors, initially at 10 μM concentration, limiting treatment to 30 min, a period avoiding large-scale apoptosis. We included the known commercially available SHIP2 inhibitor AS1949490 (**7**).^14^ We blotted for pAkt S473 and total Akt. Initial experiments revealed little effect of purpurogallin (**10**), galloflavin (**11**), estramustine phosphate (**12**), pentahydroxyflavone (**13**), or biphenyl 2,3′,4,5′,6-pentakisphosphate (**1**), but revealed a notable effect for AS1949490 (**7**). Repeat experiments with purpurogallin (**10**) and AS1949490 (**7**) showed that AS1949490 (**7**) reduced Akt phosphorylation (isoform unresolved) in a dose-dependent manner (Figure 9B) and markedly reduced cell survival (Figure 9D). While purpurogallin (**10**) was without marked effect on Akt phosphorylation (at 30 min), it abrogated the enhancement of cell survival at 24 h afforded by Il-6, reducing survival to control levels (Figure 9B,C). These data do not fit a simple model of PtdIns(3,4,5)P_3_ as an exclusive cell survival signal in MM1 cells since elevation of PtdIns(3,4,5)P_3_ might be expected to increase Akt phosphorylation.^14^ Rather, we note evidence that different Akt isoforms are differentially activated in a spatiotemporal context by either and/or both PtdIns(3,4,5)P_3_ and PtdIns(3,4)P_2_,^33^ reflecting direct activation of Akt by PtdIns(3,4)P_2_^34^ and correlation of PtdIns(3,4,5)P_3_ and PtdIns(3,4)P_2_ levels, respectively, with phosphorylation of Akt on Thr308 and Ser473.^35^ Indeed, in the context of PDGF signaling in NIH 3T3 cells, AS1949490 (**7**) reduced PtdIns(3,4)P_2_ levels specifically in the plasma membrane, reducing recruitment of Akt2 and sorting of PtdIns(3,4)P_2_ to early endosomes.^33^ Similarly and specifically in the context of multiple myeloma, pan-SHIP1/2 inhibitors have been shown to kill multiple myeloma cells and in a SHIP2-expressing breast cancer cell reduced cell numbers.^36^ Consequently, our data are consistent with more comprehensive studies and reviews of SHIP1/2 function in immune cell context that point to therapeutic utility of SHIP1/2 inhibitors.^4,11,36,37^

## Conclusions

By use of orthogonal assays and a powerful fluorescent active-site ligand displacement strategy,^16,19^ we have defined a regulatory site on the catalytic domain of SHIP2. Occupancy of this site by 1,2,4-dimer (**5**) inhibits catalytic activity against Ins(1,3,4,5)P_4_ in an uncompetitive manner. The ligand coordinates an aspartate residue on the L4 loop that is part of a network of intramolecular interactions between the C2-domain and the phosphatase domain.^18^ The shallow pocket occupied by the ligand provides a potential target for fragment-based screening approaches for novel inhibitors of SHIP2. These might need to be structurally extended in nature in line with the 1,2,4-dimer (**5**) and most ideally without any phosphate groups. The structurally validated approach of use of fluorescent active site ligands as a screening tool should assist facile robotic screening of larger compound libraries for modulators of the SHIP2 function. Extension of the screen to multidomain SHIP2 protein should readily allow identification of allosteric modulators whose binding lies on other domains of the protein. Quite separately, the discovery of GPP (**19**) and IPP (**20**) as potential ligands and substrates of SHIP2 is tantalizing, given the role of inactivating mutations of SHIP2 in opsismodysplasia, a skeletal chondroplasia,^38^ and the use of bisphosphonates, which target isoprenoid metabolism to prevent bone resorption.^39^

## Experimental Section

### Chemicals

Ins(1,3,4,5)P_4_ was prepared according to ref (40). Ins(1,3,4)P_3_ and Ins(1,4,5)P_3_ were obtained from AG Scientific as K^+^ salts. Ins(3,4,5)P_3_ was obtained from SiChem as Na^+^ salt. All biphenyl phosphates and related compounds were prepared as described^23,24^ or in a very similar fashion. AS 1949490 (**7**) was obtained from Tocris. Sodium pyrophosphate, geranyl pyrophosphate, and isopentenyl pyrophosphate were obtained from Sigma-Aldrich. Di-C_8_-PtdIns(3,4,5)P_3_ was obtained from Echelon Biosciences.

All compounds biologically evaluated had >95% purity, as determined by reverse-phase hplc on a 4.6 × 250 mm Phenomenex Synergi Hydro-RP column eluted isocratically at 1 ml.min^–1^ with a solvent mixture comprising 4 mM tetrabutylammonium hydroxide in 70/30, v/v, 50 mM NaH_2_PO_4_/(acetonitrile/MeOH/water, 40/50/10, v/v/v). Samples, 10 μL of 10 μM compound, were injected, and peaks were detected with a Jasco FP-950 fluorescence detector set at Ex 285 nm, Em 320 nm and gain 10.

### Protein Purification

An expression clone of human *INPPL1cd* (SHIP2 catalytic domain, residues 419–832 in the vector pNIC-MBP) was obtained from Source BioScience (clone accession TC124029). Recloning, expression, and purification were performed as described.^16^

### Docking Simulations

Docking simulations were performed as described^19^ with the SHIP2 catalytic domain (SHIP2cd) as an inflexible receptor and 2-FAM-InsP_5_ as a flexible ligand.

### Crystallization, Data Collection, and Refinement

Crystals were grown in 0.5 μL sitting drops at 16 °C, equilibrated against a reservoir containing 50 μL of the precipitant (0.17 M ammonium sulfate, 25.5% PEG 4000, 15% glycerol). Protein at 10 mg/mL was mixed with an equal volume of the precipitant. Single crystals were soaked for 6 min in a 1 μL drop containing 0.17 M ammonium sulfate, 25.5% PEG 4000, 15% glycerol, and 6.8 mM 5,5′-ethane-1,2-diylbis(oxy)*bis*(benzene-1,2,4-trisphosphate) (1,2,4-dimer) (**5**), and flash-frozen in liquid nitrogen. X-ray diffraction data were collected on beamline I03 at the Diamond Light Source (Oxford). Molecular replacement and refinement were carried out using Phenix,^41^ and manual rebuilding was performed using Coot.^42^ Automated identification and placement of ligands was carried out using the ligand_identification facility in Phenix.^29,30^

### Fluorescence Polarization Screening of SHIP2

The method used was as described in ref (19) using a 20 mM HEPES pH 7.3, 50 mM KCl, 1 mM EDTA buffer. Screening assays were performed by hand at 100 nM SHIP2, 5 nM 2-FAM-InsP_5_, and 10 μM screen compound using NCI Diversity set II, Developmental Therapeutics Program NCI/NIH, and the NCI Approved Oncology Drug (AOD) Set screens in 96-well black plates on a ClarioStar (BMG Ltd) plate reader. Displacement assays were performed using 100 nM SHIP2cd and 2 nM 2-FAM-InsP_5_ 1 in 384-well black plates. Polarization values were fitted to a four-parameter logistic in GraphPad Prism v6.0 after export from the MARS software of the plate reader.

### Phosphate Release Assays

Enzyme reactions containing 100 nM SHIP2cd, 250 μM Ins(1,3,4,5)P_4_, and inhibitor (100 nM–100 μM) in 20 mM HEPES pH 7.3, 50 mM KCl, 1 mM EDTA buffer were incubated for 20 min at 30 °C. In a 96-well plate, 10 μL of the enzyme reaction mixture was mixed with 10 μL of the color reagent [4 parts 1.5% w/v ammonium molybdate in a 5.5% v/v sulfuric acid solution; 1 part 10.8% w/v iron(II) sulfate solution]. After 10 min incubation at room temperature, absorbance at 700 nm was measured using a Hidex Sense (LabLogic Systems, UK) microplate reader. Measurements of background buffer absorbance were subtracted from the absorbance values and the resulting values converted to phosphate by reference to a standard curve constructed with 1–50 μM KH_2_PO_4_. Results were plotted and fitted to a four-parameter logistic using GraphPad Prism v6.0. For analysis of the modality of enzyme inhibition by 1,2,4-dimer (**5**), assays were performed in the same buffer with 100 nM SHIP2cd, varying Ins(1,3,4,5)P_4_, and inhibitor. Reactions were performed for 35 min at 30 °C. Results were fitted globally to a mixed model in GraphPad Prism version 6 for analysis of kinetic parameters and the thermodynamic cooperativity factor.^28^

### HPLC Assay of Active-Site Binding of 2-FAM-InsP_5_

Assays to determine whether SHIP2 can dephosphorylate 2-FAM-InsP_5_ were performed by incubating 100 nM SHIP2cd with 50 μM 2-FAM-InsP_5_ in 20 mM HEPES pH 7.3, 1 mM EDTA buffer, pH 7.3, overnight at 25 °C. Aliquots (20 μL) of the reaction products were diluted to 50 μL, and a 20 μL sample was analyzed by HPLC on a 3 mm i.d. A CarboPac PA200 column was eluted with methanesulfonic acid.^43^ Substrates and products were detected with a Jasco FP-920 fluorescence detector set at Ex485 nm, 10 nm band pass; Em520 nm, 10 nm band pass, and a gain of 10. Data were exported from ChromNav v1.0 software of the Jasco HPLC machine as ASCII files and redrawn with GraphPad Prism v6.0.

### HPLC Assay of 5-Phosphatase Activity of SHIP2

Enzyme reactions from the phosphate release assays performed as described above with 250 μM Ins(1,3,4,5)P_4_ were also analyzed by HPLC. Reactions of 10 μL were stopped by the addition of EDTA to 5 mM, diluted with water to 50 μL, and a 20 μL aliquot injected onto a 3 mm i.d. CarboPac PA200 column (Dionex, UK) eluted with a gradient of methanesulfonic acid. Inositol phosphates were detected by postcolumn complexation with ferric nitrate.^43^ Peak areas were integrated in the ChromNav v2.0 software of the Jasco HPLC machine. For verification of the reaction catalyzed by SHIP2, inositol trisphosphates were spiked into reaction products to a final (injected) concentration of 50–200 μM (Figure S1). For reproduction of chromatograms, data were exported from the ChromNav2 software as x,y data files and redrawn in GraphPad Prism v6.0.

### Biological Methods

Antiphosphorylated and pan Akt antibodies were purchased from Cell Signalling Technology (Cambridge, MA, USA). Interleukin-6 (Il-6) was purchased from Miltenyi Biotec (Auburn, CA, USA).

The authenticated multiple myeloma-derived cell line MM1 was obtained from the European Collection of Cell Cultures and was cultured in RPMI 1640 medium supplemented with 10% foetal bovine serum, penicillin, and streptomycin (all obtained from Invitrogen, Paisley, UK).^44^

Cell viability was determined after 24 h using Cell Titre GLO (Promega, Southampton, UK).^31^ Data were normalized to vehicle controls. All data points are represented as the mean with s.e.

### Western Immunoblotting

Sodium dodecyl sulphate polyacrylamide gel electrophoresis and western blot analyses were performed, as described previously.^44^

### Data Analysis

Dose–response relationships were fitted to Hill equations (GraphPad Prism, version 6), from which IC_50_ values were obtained.

## Supporting Information Available

Binding of FAM/FITC-InsPs to SHIP2cd, X-ray data collection and refinement statistics, top 10 ranked hits from Phenix ligand identification, diversity Set II Compound Library Plate Map JMC.xls: NSC ids of compounds in NCI Diversity Set II tested in this study, structures of drug-like compounds tested in this study, HPLC assay of Ins(1,3,4,5)P_4_-directed 5-phosphatase activity, fitting of dose–response of inhibition of SHIP2 5-phosphatase activity by biphenyl phosphates and related compounds, fitting of dose–response of inhibition of SHIP2 5-phosphatase activity by compounds from NCI-diversity and AOD sets, electron density maps for 1,2,4-dimer (**5**) in its complex with SHIP2cd (PDB 6SQU), comparison of binding sites for SHIP1/2 allosteric ligands, substrate specificity of SHIP2 for inositol phosphates and biphenyl phosphates, and fit of geranyl pyrophosphate (**19**) to active site residual electron density in the crystal structure of the SHIP2cd complex with 1,2,4-dimer (PDB 6SQU) (PDF)
Molecular docking for receptor: SHIP2-monomer (PDB)
Molecular docking for 2-FAM-InsP_5_:pose-P3 (PDB)
Molecular docking for 2-FAM-InsP_5_: pose-P4 (PDB)
NSC identifiers and plate map of NCI Diversity set II compound library (XLS)
SMILES file and associated data for compounds described, including 1,2,4-dimer ligand of SHIP2cd: PDB 6SQU (CSV)

## Supplementary Material

jm0c01944_si_001.pdf

jm0c01944_si_002.pdb

jm0c01944_si_003.pdb

jm0c01944_si_004.pdb

## Abbreviations

1,2-dimer: 4,4′-ethane-1,2-diylbis(oxy)*bis*(benzene-1,2-bisphosphate)
1,2,4-dimer: 5,5′-ethane-1,2-diylbis(oxy)*bis*(benzene-1,2,4-trisphosphate)
2-FAM-InsP_5_: 2-*O*-(2-(5-fluoresceinylcarboxy)-aminoethyl)-*myo*-inositol 1,3,4,5,6-pentakisphosphate (triethylammonium salt)
3AC: 3α-aminocholestane
6,6′-F_2_-BiPh(3,3′,4,4′)P_4_: 6,6′-difluoro biphenyl-3,3′,4,4′-tetrakisphosphate
Bz(1,2)P_2_: benzene 1,2-bisphosphate
Akt: protein kinase B
Bz(1,2,4)P_3_: benzene 1,2,4-trisphosphate
Bz(1,2,4,5)P_4_: benzene 1,2,4,5-tetrakisphosphate
BiPh(2,3′,4,5′,6)P_5_: biphenyl-2,3′,4,5′,6-pentakisphosphate
BiPh(2,2′,4,4′,5,5′)P_6_: biphenyl-2,2′,4,4′,5,5′-hexakisphosphate
BiPh(3,3′,4,4′,5,5′)P_6_: biphenyl-3,3′,4,4′,5,5′-hexakisphosphate
EC_50_: half-maximal effective concentration
EDTA: ethylenediamine tetra-acetic acid
GPP: geranyl pyrophosphate
HEPES: 4-(2-hydroxyethyl)-1-piperazineethane sulfonic acid
His: histidine
HPLC: high-pressure liquid chromatography
IC_50_: half-maximal inhibitory concentration
IL-6: interleukin 6
INPP5A: type I inositol 5-phosphatase
INPP5B: type II inositol 5-phosphatase
INPP5E: inositol polyphosphate 5-phosphatase E
Ins(4,5)P_2_: 1 *d*-*myo*-inositol 4,5-bisphosphate
Ins(1,3,4)P_3_: 1d-*myo*-inositol 1,3,4-trisphosphate
Ins(1,4,5)P_3_: 1d-*myo*-inositol 1,4,5-trisphosphate
Ins(3,4,5)P_3_: 1d-*myo*-inositol 3,4,5-trisphosphate
Ins(1,2,4,5)P_4_: 1d-*myo*-inositol 1,2,4,5-tetrakisphosphate
Ins(1,3,4,5)P_4_: 1d-*myo*-inositol 1,3,4,5-tetrakisphosphate
IP_3_R: Ins(1,4,5)P_3_ Receptor
IPP: isopentenyl pyrophosphate
OCRL-1: Lowe oculocerebrorenal syndrome protein (INPP5F)
pAkt: phosphorylated Akt
PDGF: platelet-derived growth factor
PtdIns4P: phosphatidyl 1d-*myo*-inositol 4-monophosphate
PtdIns(3,4)P_2_: phosphatidyl 1d-*myo*- inositol 3,4-bisphosphate
PtdIns(3,4,5)P_3_: phosphatidyl 1d-*myo*-inositol 3,4,5-trisphosphate
SHIP1: SH2 domain-containing inositol 5-phosphatase type 1
SHIP2: SH2 domain-containing inositol 5-phosphatase type 2
SKIP1 (INPP5K): inositol polyphosphate 5-phosphatase K
SYNJ1: synaptojanin-1
SYNJ2: synaptojanin-2
