# Supplementary material for: Allosteric Site on SHIP2 Identified Through Fluorescent Ligand Screening and Crystallography: A Potential New Target for Intervention
Source: J Med Chem. 2021 Mar 16;64(7):3813–26. doi: 10.1021/acs.jmedchem.0c01944 (PMC7610569; doi:10.1021/acs.jmedchem.0c01944)
Supplement: Supplementary file 1 — jm0c01944_si_001.pdf [file jm0c01944_si_001.pdf]

Supporting Information

Allosteric site on SHIP2 identified through fluorescent ligand screening and crystallography: a potential new target for intervention

Hayley Whitfield[†], Andrew M Hemmings[§*], Stephen J Mills[‡], Kendall Baker[†], Gaye White[†], Stuart Rushworth[¶], Andrew M Riley[‡], Barry V L Potter[‡*] and Charles A Brearley[†*]

[†]School of Biological Sciences, University of East Anglia, Norwich Research Park, Norwich NR4 7TJ, UK.

[§]School of Chemistry, University of East Anglia, Norwich Research Park, Norwich NR4 7TJ, UK.

[¶]Department of Molecular Haematology; Norwich Medical School; University of East Anglia; Norwich, United Kingdom.

[‡]Medicinal Chemistry & Drug Discovery, Department of Pharmacology, University of Oxford, Mansfield Road, Oxford OX1 3QT, UK

Corresponding Authors

*Tel; +44 1603 592197. Email: c.brearley@uea.ac.uk

*Tel; +44 1865 271945. Email: barry.potter@pharm.ox.ac.uk

*Tel; +44 1603 592259. Email: a.hemmings@uea.ac.uk

ORCID

Charles A Brearley: 0000-0001-6179-9109

Barry V L Potter: 0000-0001-3255-9315

Andrew M Hemmings: 0000-0003-3053-3134

Andrew M Riley: 0000-0001-9003-3540

Stuart Rushworth: 000-0002-3711-7558

Author Contributions

C.A.B., K.B., A.M.H., S.J.M., A.M.R. S.R., G.W. and H.W. and performed experiments. C.A.B., A.M.H., and B.V.L.P. designed the study. C.A.B., A.M.H., B.V.L.P., A.M.R. S.R., G.W. and H.W. wrote the manuscript.

Funding enabling this study in support of H.W. was obtained by C.A.B. (BBSRC BB/N002024/1 with contribution from AB Vista), K.B. was supported by AB Vista. B.V.L.P. is a Wellcome Trust Senior Investigator (Grant 101010). We acknowledge Diamond Light Source for time on beamline I03 under proposal MX13467.

TABLE OF CONTENTS

Table S1: Binding of FAM/FITC-InsPs to SHIP2cd

Table S2: X-ray data collection and refinement statistics

Table S3: Top 10 ranked hits from Phenix ligand identification

Table S4: Diversity Set II Compound Library Plate Map JMC.xls: NSC ids of compounds in NCI Diversity Set II tested in this study.

Figure S1: Structures of drug-like compounds tested in this study

Figure S2: HPLC assay of Ins(1,3,4,5)P_4-directed 5-phosphatase activity

Figure S3: Fitting of dose-response of inhibition of SHIP2 5-phosphatase activity by biphenyl phosphates and related compounds

Figure S4: Fitting of dose-response of inhibition of SHIP2 5-phosphatase activity by compounds from NCI-diversity and AOD sets

Figure S5: Electron density maps for 1,2,4-dimer (**5**) in its complex with SHIP2cd (PDB 6SQU).

Figure S6: Comparison of binding sites for SHIP1/2 allosteric ligands.

Figure S7: Substrate specificity of SHIP2 for inositol phosphates and biphenyl phosphates

Figure S8: Fit of geranyl pyrophosphate (**19**) to active site residual electron density in the crystal structure of the SHIP2cd complex with 1,2,4-dimer (PDB 6SQU)

Docking files: pdb files for receptor (SHIP2), SHIP2-monomer.pdb; 2-FAM-InsP_5, pose-P3.pdb; pose-P4.pdb.

Table S1: Binding of FAM/FITC-InsPs to SHIP2cd

	EC_{50}[1] (nM)
2-FAM-InsP_5	57±1
5-FAM-InsP_5	27±2
2-FAM-Ins(1,4,5)P_3	781±1
1-FITC-InsP_3	410±1
2-FITC-Ins(1,4,5)P_3	611±1
scyllo-FITC-InsP_3	1018±1

[1] EC50 determined in 20mM HEPES, pH 7.3, 1mM $MgCl_2$

Table S2: X-ray data collection and refinement statistics[1]

PDB ID	6SRR	6SQU
Ligands	Apo	1,2,4-Dimer
**Data collection**		
Wavelength (Å)	0.9762	0.9763
Space group	P 1 21 1	P 1 21 1
Unit cell a,b,c (Å) α,β,γ (˚)	45.1, 62.1, 115.6 90.0, 92.4, 90.0	44.4, 60.2, 114.0 90.0, 92.8, 90.0
Resolution (Å)	42.30 - 2.45 (2.51 - 2.45)	41.35 - 2.27 (2.33 - 2.27)
Number of unique reflections	23610 (1733)	27911 (2067)
Completeness (%)	99 (100)	100 (100)
Multiplicity	4.4 (4.4)	3.4 (3.5)
Rmerge	0.096 (1.000)	0.079 (1.061)
Rpim	0.052 (0.541)	0.050 (0.661)
Rmeas	0.109 (1.141)	0.094 (1.253)
<I/sigma(I)>	9.5 (1.7)	9.6 (1.1)
CC1/2	1.00 (0.51)	1.00 (0.58)
Wilson B factor (Å^2)	59.79	49.57
**Refinement**		
Resolution range	36.28 – 2.45 (2.54 – 2.45)	40.68 – 2.27 (2.35 – 2.27)
Reflections used in refinement	23586 (2329)	27890 (2770)
Reflections used for R-free	1112 (137)	1344 (134)
R-work (%)	18.7 (28.5)	20.1 (29.7)
R-free (%)	23.7 (34.5)	25.4 (35.1)
Number of non-hydrogen atoms	4831	4781
Protein residues	600	580
RMS(bonds)	0.009	0.002
RMS(angles)	1.00	0.52
Ramachandran favoured (%)	93	96
Ramachandran outliers (%)	0.34	0.18
Rotamer outliers (%)	2.3	1.3
Average B-factor (Å^2)	80.38	66.09
**Ligand[2]**		
Occupancy	-	0.91
RSCC[2]	-	0.89
RSR[3]	-	0.17

[1]Data in brackets refers to high resolution bin. [2]RSCC= Real space correlation coefficient, [3]RSR= Real space R-factor

Table S3: Top 10 ranked hits from Phenix ligand identification

Rank	PDB Chemical ID	Ligand	No. of atoms	PubChem	CC[1]	Z-score
1	U10	Ubiquinone-10	63	5281915	0.42	1.77
2	FPP	Farnesyl pyrophosphate	24	445713	0.61	1.76
3	ARG	Arginine	12	52941769	0.64	1.66
4	1PG	Pentaethylene glycol monomethyl ether	17	90255	0.63	1.52
5	TDP	Thiamin diphosphate	26	5431	0.57	1.45
6	1PE	Pentaethylene glycol	16	62551	0.63	1.36
7	NBN	N-butyl isocyanide	6	76008	0.77	1.34
8	NIO	Nicotinic acid	9	938	0.69	1.22
9	BEZ	Benzoic acid	9	243	0.67	1.17
10	POP	Pyrophosphate	9	4995	0.67	1.16

[1]CC - Local correlation coefficient

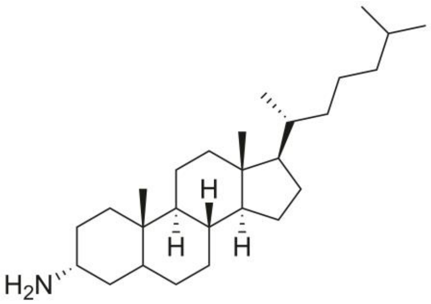

3α-aminocholestane (3-AC, **6**)

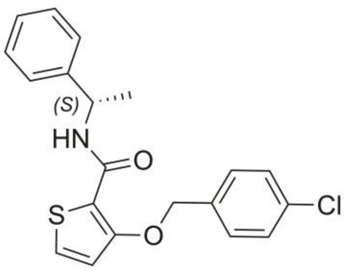

AS1949490 (**7**)

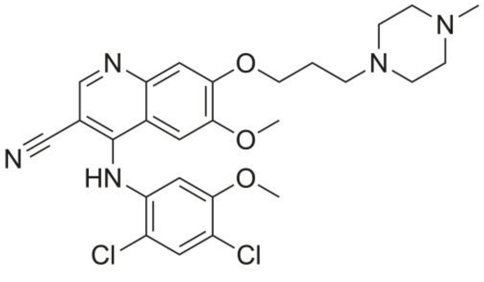

bosutinib (**8**)

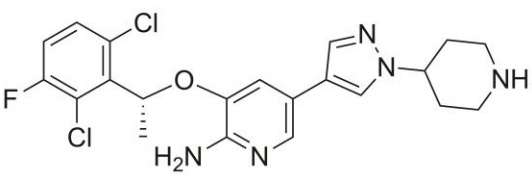

crizotinib (**9**)

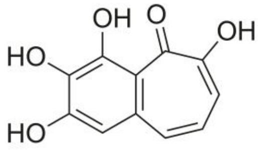

purpurogallin (**10**)

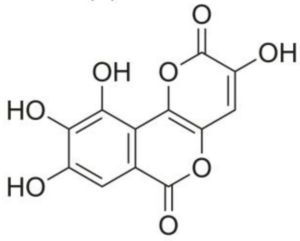

galloflavin (**11**)

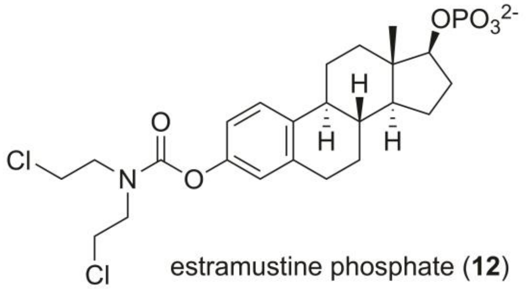

estramustine phosphate (**12**)

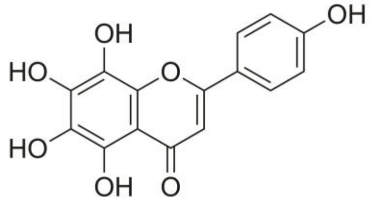

5,6,7,8,4′-pentahydroxyflavone (**13**)

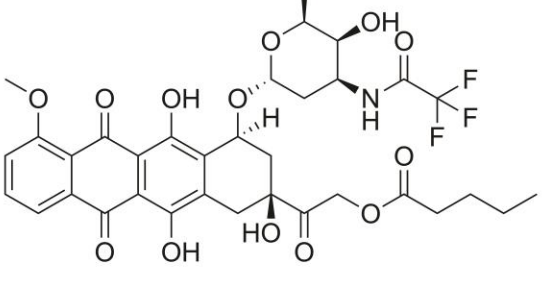

valrubicin (**14**)

Figure S1: Structures of drug-like compounds tested in this study

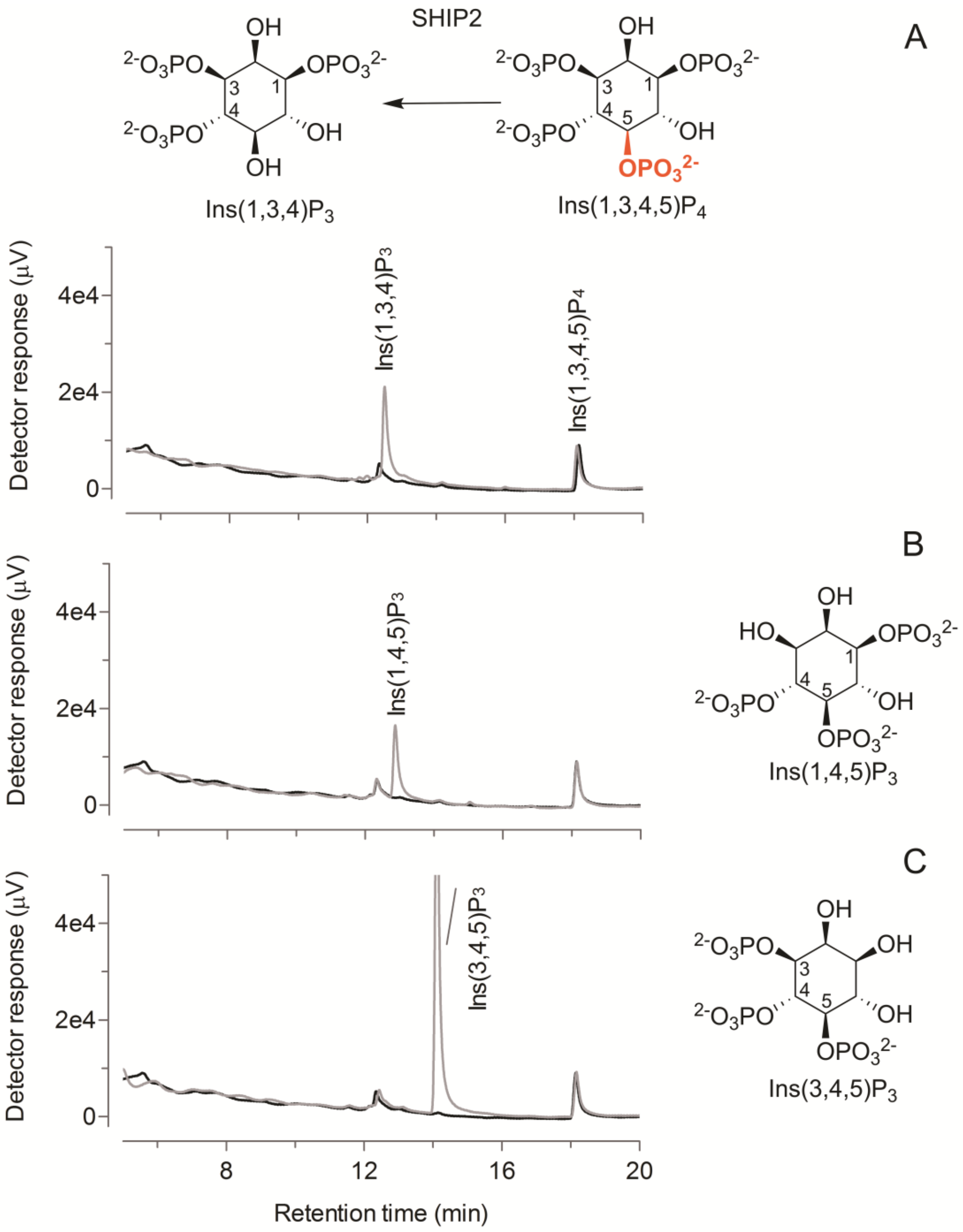

Figure S2. HPLC assay of Ins(1,3,4,5)P_4-directed SHIP2 5-phosphatase activity. A) reaction catalyzed by SHIP2, and HPLC of products of reaction of SHIP2cd (black trace, duplicated in B and C. A), B) and C) additionally show reaction products that were subsequently spiked with Ins(1,3,4)P_3, (A), Ins(1,4,5)P_3 (B) or Ins(3,4,5)P_3 (C) (gray traces). The product of dephosphorylation co-elutes with Ins(1,3,4)P_3.

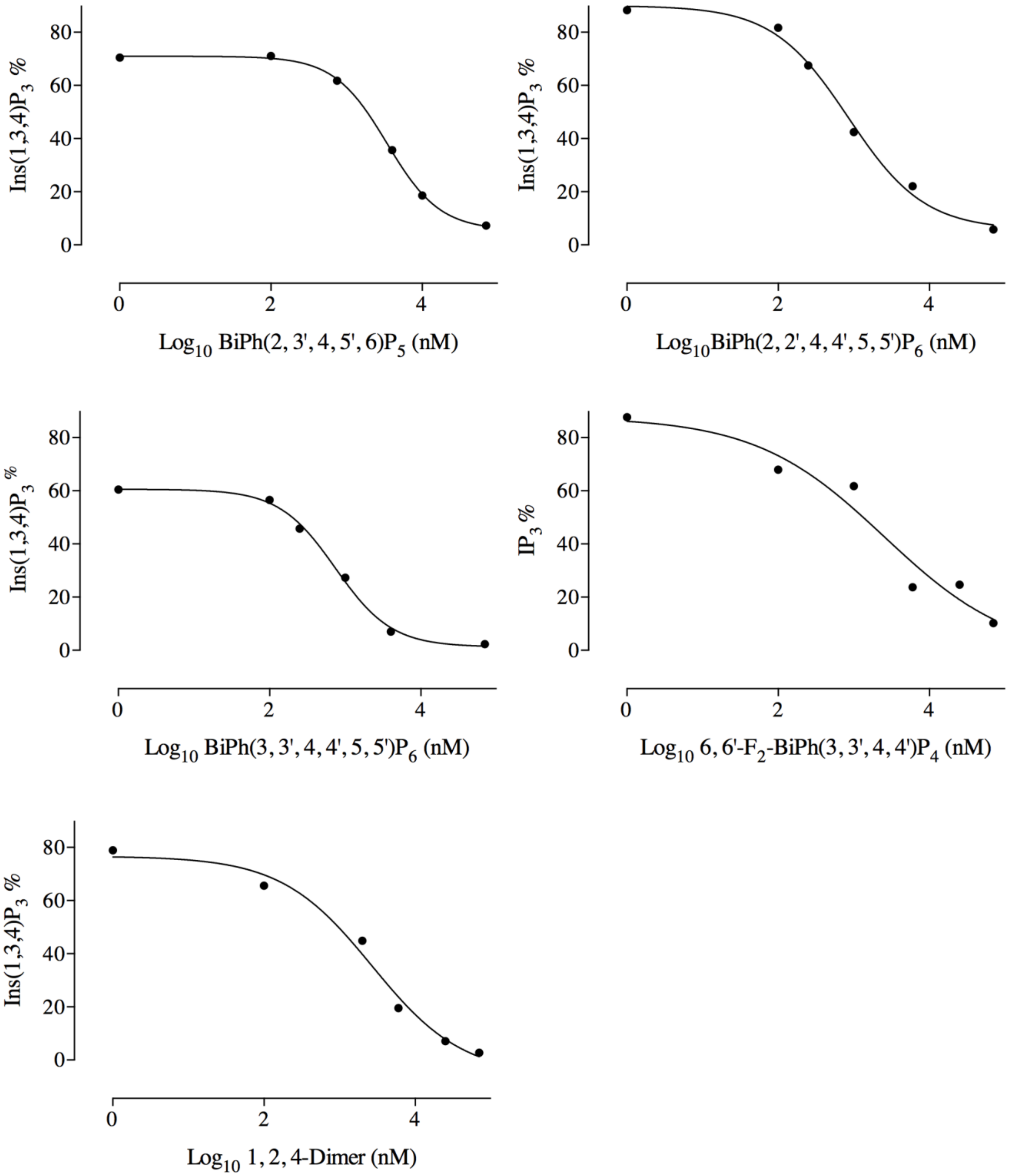

Figure S3. Dose-response of inhibition of SHIP2 5-phosphatase activity by biphenyl phosphates and related compounds. BiPh(2,3′,4,5′,6)P_5 (**1**), BiPh(2,2′,4,4′,5,5′)P_6 (**2**), BiPh(3,3′,4,4′,5,5′)P_6 (**3**), 6,6′-F_2-BiPh(3,3′,4,4′)P_4 (**4**), 1,2,4-dimer (**5**). Inositol phosphate reaction products were analysed by HPLC. Conversion of Ins(1,3,4,5)P_4 to Ins(1,3,4)P_3 was determined from integrated peak areas and data fitted to a 4-parameter logistic.

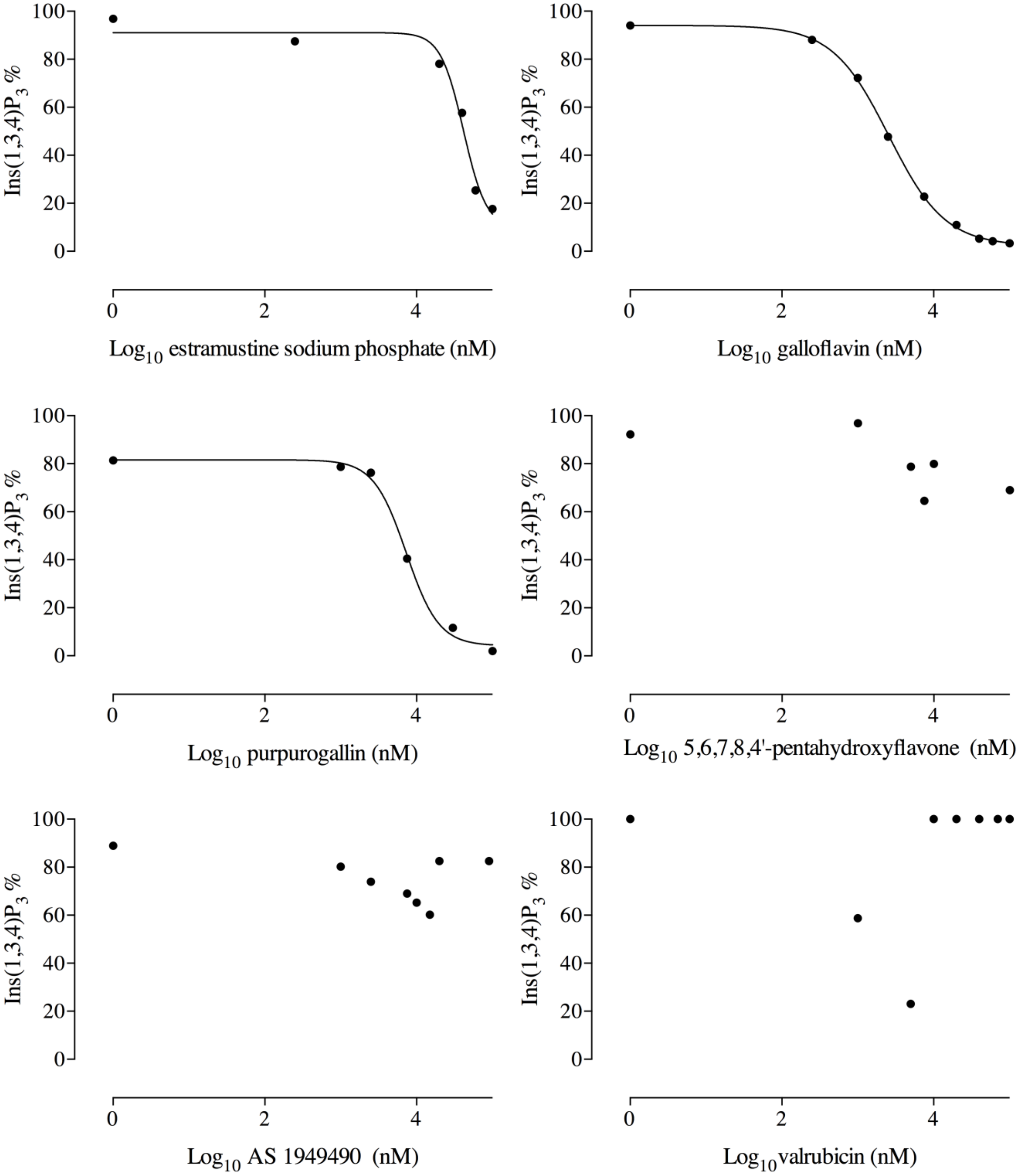

Figure S4. Dose-response of inhibition of SHIP2 5-phosphatase activity. Estramustine sodium phosphate (**12**), galloflavin (**11**), purpurogallin (**10**), 5,6,7,8,4'-pentahydroxyflavone (**13**), AS1949490 (**7**) and valrubicin **(14).** Inositol phosphate reaction products were analysed by HPLC. Conversion of Ins(1,3,4,5)P_4 into Ins(1,3,4)P_3 was determined from integrated peak areas and data fitted to a 4-parameter logistic.

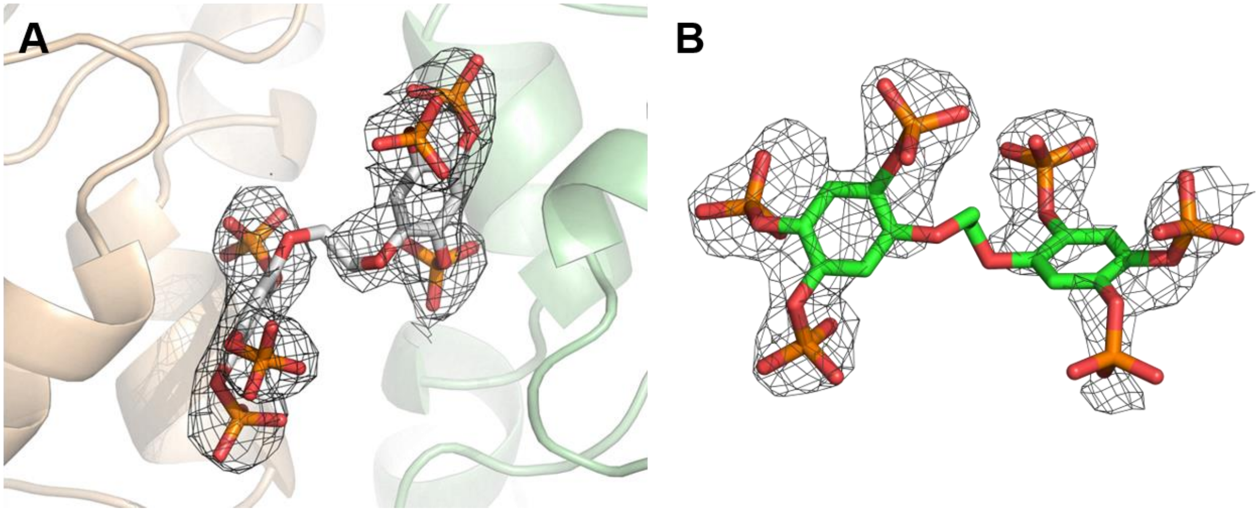

Figure S5. Electron density maps for 1,2,4-dimer (**5**) in its complex with SHIP2cd (PDB 6SQU). A) $2m|F_o| - D|F_c|$ difference Fourier electron density map contoured at 1.0σ (grey hatching) for the inhibitor (carbons in white). The view is approximately along the pseudo 2-fold axis relating protein monomers in the asymmetric unit. Individual, protein monomers are shown in wheat (monomer B) and green (monomer A). B) Omit map. $m|F_o| - D|F_c|$ difference Fourier electron density omit map contoured at 2.5σ (grey hatching). The refined coordinate of 1,2,4-dimer (carbons shown in green) are shown overlayed.

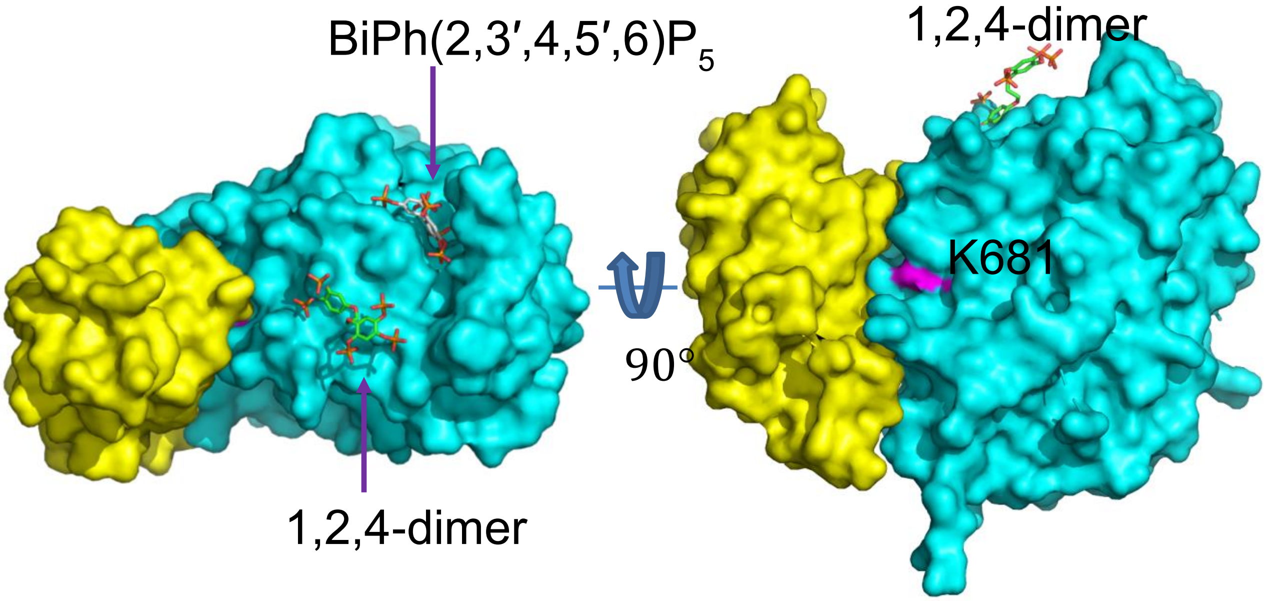

Figure S6. **Comparison of binding sites for SHIP1/2 allosteric ligands**. Orthogonal views of a molecular surface representation of SHIP1 (PDB entry 6DLG) colored by domain (phosphatase domain in cyan and C2 domain in yellow) with molecular surface contribution of residue K681 colored magenta. K681 forms part of the binding pocket for the allosteric inhibitor ZPR-MN100 [26]. Overlaid is the equivalent position of the allosteric SHIP2 inhibitor 1,2,4-dimer (**5**) (PDB 6SQU, carbons in green). The position of the competitive inhibitor BiPh(2,3′,4,5′,6)P_5 (**1**) (PDB 4A9C, grey carbons) is shown for reference.

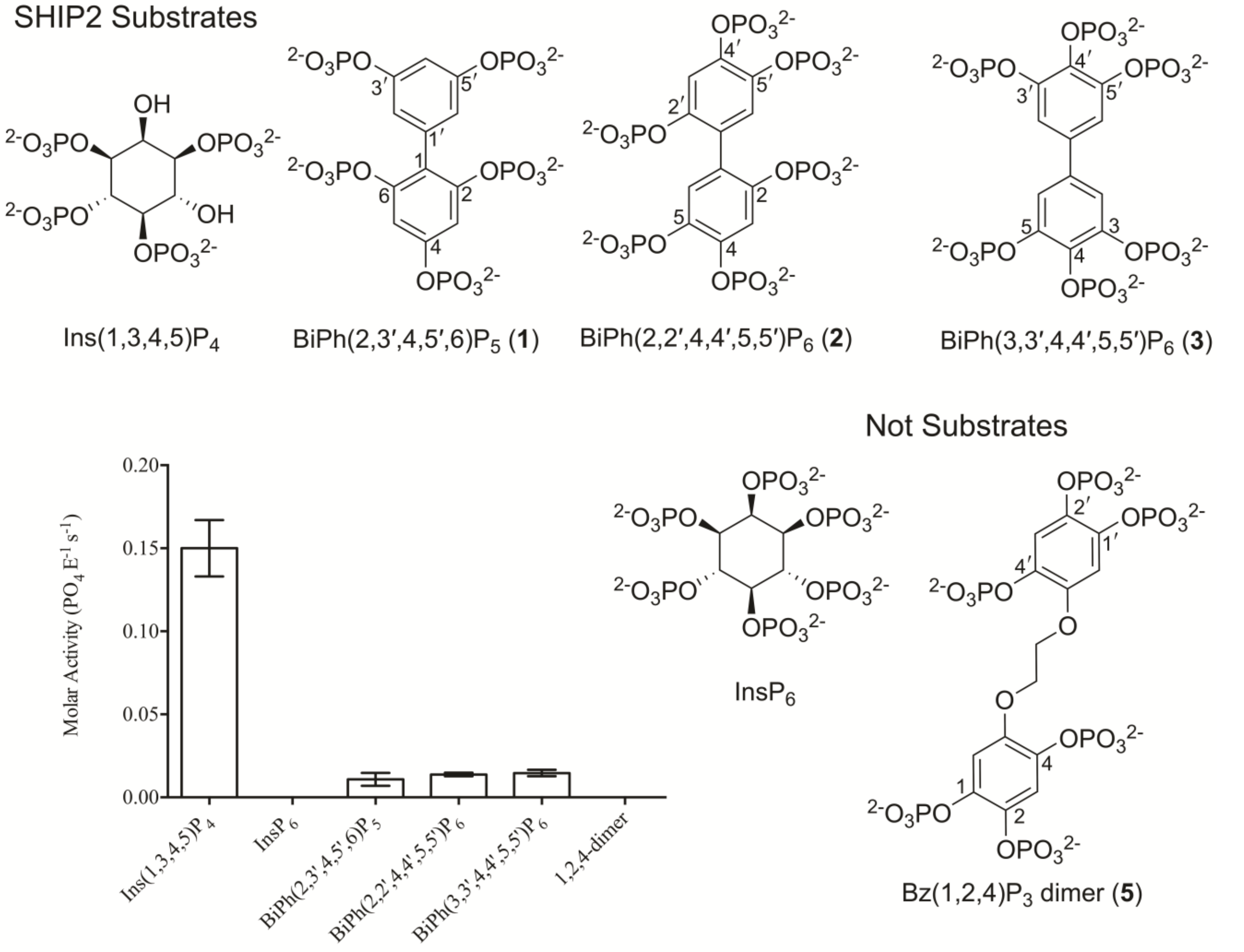

Figure S7. Substrate activity of SHIP2 towards inositol phosphate, biphenyl phosphate and related compounds. Phosphate release from Ins(1,3,4,5)P_4 and InsP_6 assayed with 1 µM SHIP2cd; biphenyl and related compounds with 10 µM SHIP2cd (mean and s.d.).

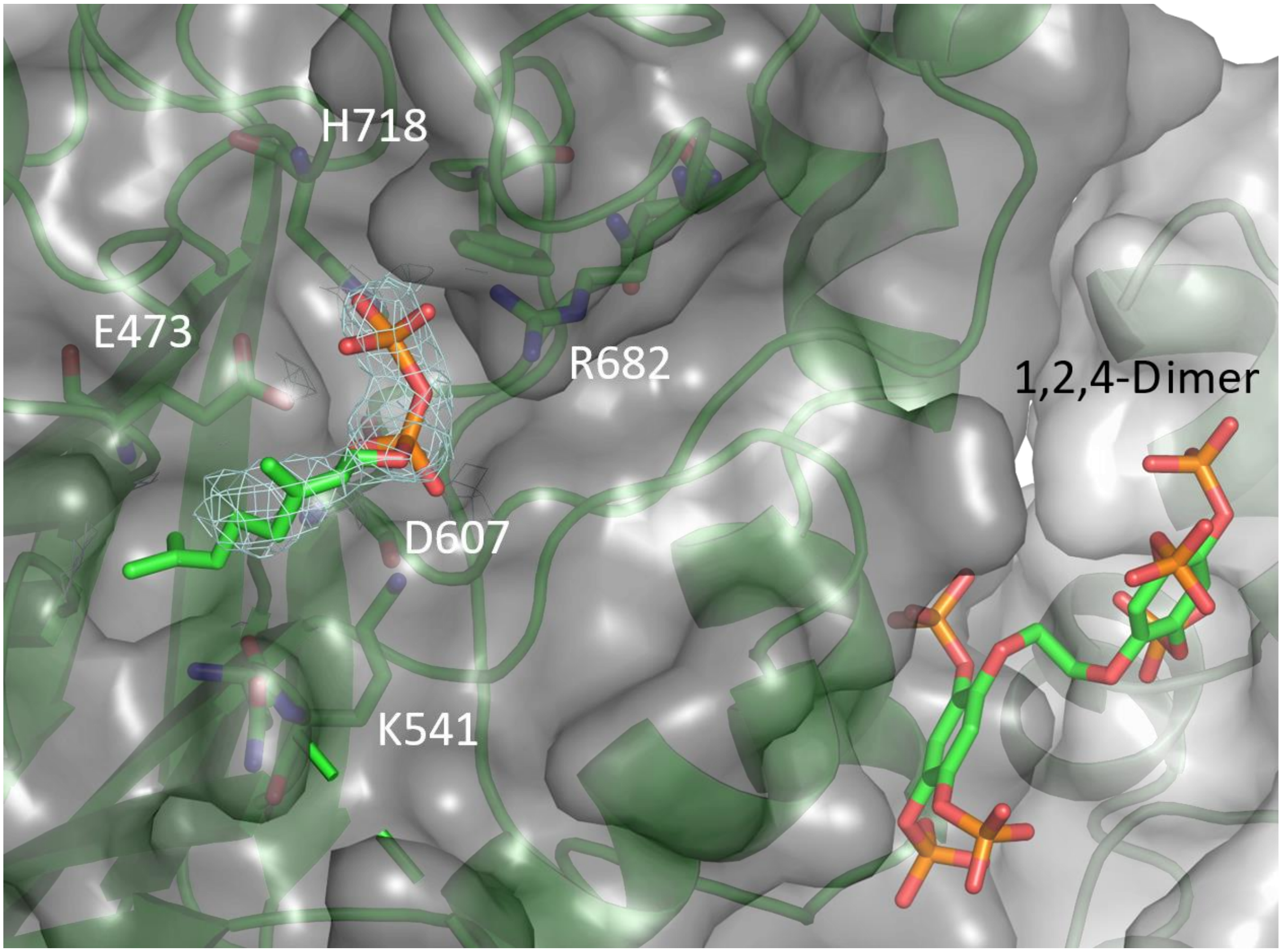

Figure S8. Fit of geranyl pyrophosphate (19) to active site residual electron density in the crystal structure of the SHIP2cd complex with 1,2,4-dimer (PDB 6SQU). The SHIP2cd dimer is shown in cartoon format (green) with a semi-transparent molecular surface. Selected active site resides are shown in stick format and labeled, as is 1,2,4-dimer shown binding at an allosteric site. Residual difference electron density (pale blue hatching; $2m|F_o| - D|F_c|$) contoured at 2.5σ is seen in the active site overlaying the docked position of farnesyl diphosphate (stick format) as determined by the ligand_fit method of Phenix.
